# The molecular mechanism of actions and clinical utilities of tumor infiltrating lymphocytes in gastrointestinal cancers: a comprehensive review and future prospects toward personalized medicine

**DOI:** 10.3389/fimmu.2023.1298891

**Published:** 2023-11-24

**Authors:** Moein Piroozkhah, Yasaman Gholinezhad, Mobin Piroozkhah, Elahe Shams, Ehsan Nazemalhosseini-Mojarad

**Affiliations:** ^1^ Basic and Molecular Epidemiology of Gastrointestinal Disorders Research Center, Research Institute for Gastroenterology and Liver Diseases, Shahid Beheshti University of Medical Sciences, Tehran, Iran; ^2^ School of Medicine, Shahid Beheshti University of Medical Sciences, Tehran, Iran; ^3^ School of Medicine, Tehran University of Medical Sciences, Tehran, Iran; ^4^ Gastroenterology and Liver Diseases Research Center, Research Institute for Gastroenterology and Liver Diseases, Shahid Beheshti University of Medical Sciences, Tehran, Iran

**Keywords:** gastrointestinal tract cancer, tumor-infiltrating lymphocytes, immunotherapy, immune microenvironment, immune infiltration

## Abstract

Gastrointestinal (GI) cancers remain a significant global health burden, accounting for a substantial number of cases and deaths. Regrettably, the inadequacy of dependable biomarkers hinders the precise forecasting of patient prognosis and the selection of appropriate therapeutic sequencing for individuals with GI cancers, leading to suboptimal outcomes for numerous patients. The intricate interplay between tumor-infiltrating lymphocytes (TILs) and the tumor immune microenvironment (TIME) has been shown to be a pivotal determinant of response to anti-cancer therapy and consequential clinical outcomes across a multitude of cancer types. Therefore, the assessment of TILs has garnered global interest as a promising prognostic biomarker in oncology, with the potential to improve clinical decision-making substantially. Moreover, recent discoveries in immunotherapy have progressively changed the landscape of cancer treatment and significantly prolonged the survival of patients with advanced cancers. Nonetheless, the response rate remains constrained within solid tumor sufferers, even when TIL landscapes appear comparable, which calls for the development of our understanding of cellular and molecular cross-talk between TIME and tumor. Hence, this comprehensive review encapsulates the extant literature elucidating the TILs’ underlying molecular pathogenesis, prognostic significance, and their relevance in the realm of immunotherapy for patients afflicted by GI tract cancers. Within this review, we demonstrate that the type, density, and spatial distribution of distinct TIL subpopulations carries pivotal implications for the prediction of anti-cancer treatment responses and patient survival. Furthermore, this review underscores the indispensable role of TILs in modulating therapeutic responses within distinct molecular subtypes, such as those characterized by microsatellite stability or programmed cell death ligand-1 expression in GI tract cancers. The review concludes by outlining future directions in TIL-based personalized medicine, including integrating TIL-based approaches into existing treatment regimens and developing novel therapeutic strategies that exploit the unique properties of TILs and their potential as a promising avenue for personalized cancer treatment.

## 1 Introduction

Gastrointestinal (GI) cancers are responsible for a significant portion of the global cancer burden, accounting for 26% of all new cases and 35.4% of all cancer-related deaths. Notably, six types of GI cancers, including liver, stomach, colon, esophagus, pancreas, and rectum cancers, rank among the top ten list of tumor-related mortality rates worldwide (1).

The American Joint Committee on Cancer (AJCC) and Union for International Cancer Control (UICC) established the tumor-node-metastasis classification system (TNM) to assess the histopathological parameters of tumor invasion and predict the prognosis and chemotherapy response of individuals with GI cancers (2, 3). However, despite receiving equivalent treatment, patients with similar TNM stages may experience varying clinical outcomes due to the genetic and epigenetic heterogeneity of the cancers, as evidenced by several studies (4–6). The advent of anticancer therapy, particularly the remarkable advancements in immunotherapy, has significantly transformed the treatment of cancers. Nevertheless, only a subset of GI cancer patients exhibits a favorable response to these treatments, and the underlying causes of treatment failure in other patients remain poorly understood (7, 8). Therefore, gaining a comprehensive understanding of the molecular mechanisms that drive cancer progression is critical to facilitating the development of effective prognostic and predictive biomarkers for clinical research and practice (9).

Cancer cells require a conducive microenvironment to thrive and proliferate. The tumor microenvironment (TME) comprises a dynamic and complex network of factors, including immune cells, cancer-associated fibroblasts, stroma, and blood vessels, which create a favorable milieu for tumor growth, i.e., a low oxygen concentration, an acidic pH, and immunosuppression, which are known to promote tumor development and progression (10, 11).

Tumor-infiltrating lymphocytes (TILs) are a crucial component of the TME, playing a critical role in modulating the immune response to cancer cells. TILs are thought to exert both pro-tumor and anti-tumoral effects (12), and their presence and abundance in the TME have been implicated in cancer prognosis and response to therapy in various malignancies, including breast, lung, and ovarian cancers (13–15). Considering the pivotal role of TILs in cancer development and their impact on the efficacy of various anticancer therapies, this review conducts a comprehensive assessment of the molecular mechanisms of TILs in GI cancers. Furthermore, this review evaluates the responsiveness of different therapeutic strategies currently in clinical development to TILs, providing crucial insights into their clinical utility.

## 2 Method

### 2.1 Search strategy

The authors conducted an extensive literature search to gather relevant studies on the subject. The investigation was conducted using PubMed and several high-impact journals, with diverse search terms such as “Tumor-infiltrating lymphocytes”, “T cells”, “CD8^+^”, “CD4^+^”, “CD3^+^”, “CD45RO^+^”, “FOXP3^+^”, “B cell”, “Immunoscore”, “Immunotherapy”, “Checkpoint inhibitor”, “Chemotherapy”, “Microsatellite*”, “Esophageal cancer”, “Stomach cancer”, “Gastric cancer”, “Pancreatic cancer”, “Hepatocellular cancer”, “Colorectal cancer”, “ Prognosis”, and “Survival”. The authors prioritized studies published from 2018 to 2023, and when no studies were available, older literature was included. The comprehensive search strategy ensured that the study’s findings were based on the most up-to-date and relevant available evidence.

### 2.2 Eligibility criteria

The research included studies investigating immune infiltrates in non-metastatic and metastatic GI cancer patients. The studies analyzed various oncological outcomes, including response rate and survival, while also considering patients’ clinicopathological features and molecular assessments, such as the MMR status of microsatellite stability or instability. To ensure the quality of the evidence, the researchers excluded preclinical studies, narrative reviews, editorials, letters, opinions, non-English language publications, and conference abstracts that lacked sufficient methodological details. By applying these strict inclusion and exclusion criteria, the authors aimed to provide a comprehensive analysis of high-quality studies to support their findings.

## 3 Cellular and molecular dynamics of the tumor immune microenvironment

**Figure 1:**
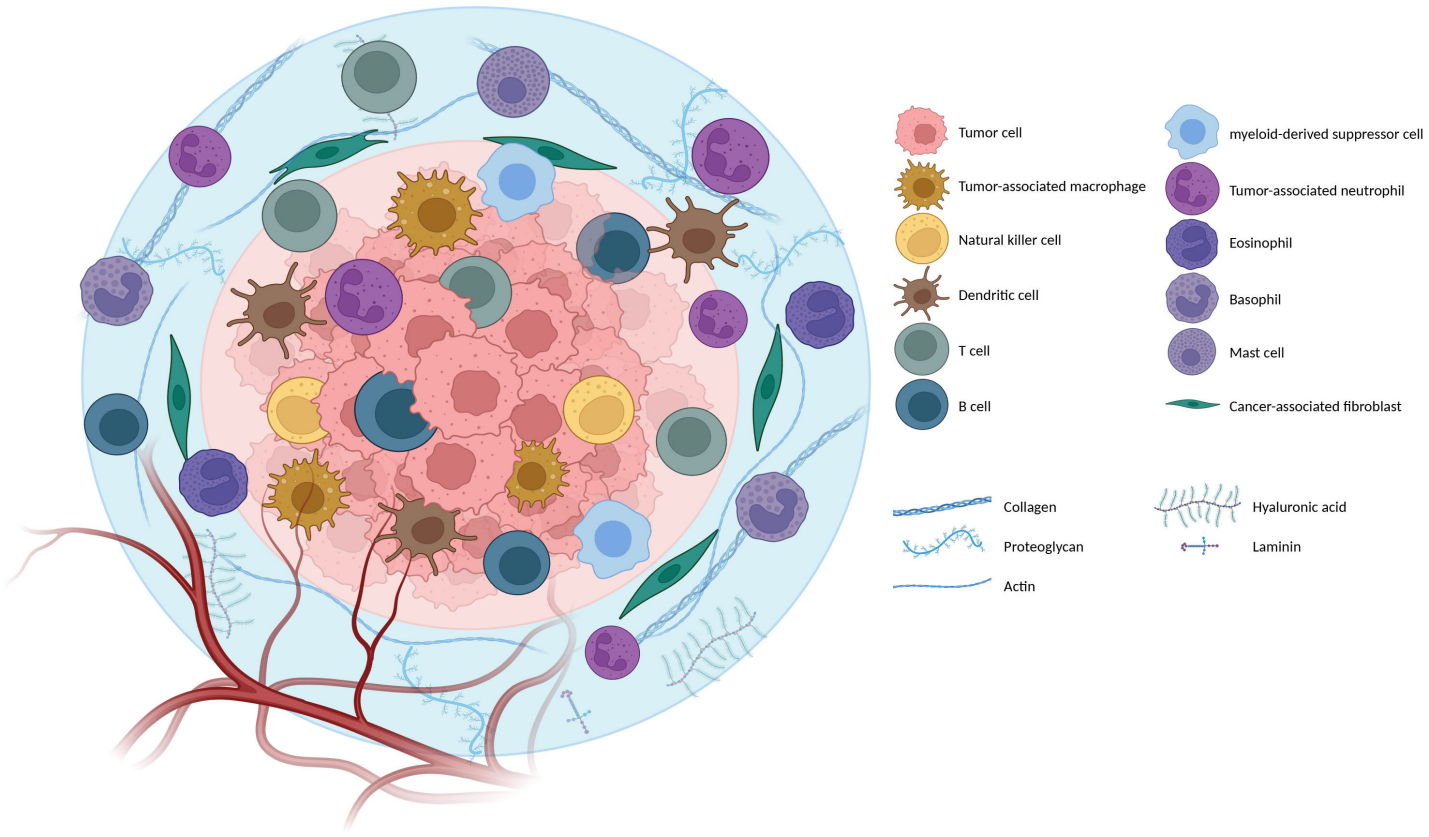
Orchestra of the Tumor microenvironment (TME): The tumor microenvironment is the environment surrounding a tumor inside the body. It includes immune cells, the extracellular matrix, blood vessels, and other cells, like fibroblasts; different TME cells and components are mentioned in the figure (Created with BioRender.com).

The tumor immune microenvironment (TIME) is an integral component of the TME. It comprises a complex and dynamic network of immune cells and their associated molecules that engage in intricate interactions with tumor cells. The TIME is composed of a diverse array of immune cell types, including tumor-associated macrophages, myeloid-derived suppressor cells (MDSCs), tumor-associated neutrophils, mast cells, natural killer (NK) cells, dendritic cells (DCs), and T and B lymphocytes, that infiltrate cancerous tissues (16). The functions of these immune cells in the TIME are tightly linked to their inherent properties and the molecules they express, such as cytokines or inhibitory ligands, and play a crucial role in tumor evolution and growth (17, 18). Hence, the primary focus of recent translational clinical research is to enhance patients’ immune systems to enhance their ability to combat and eliminate cancer cells effectively. However, it is essential to note that, similar to the two-faced ancient Roman god *Janus*, immune system cells can exhibit dual roles, either mounting a protective antitumor response or inadvertently facilitating cancer progression. This duality largely depends on the composition of immune cell infiltrates within the tumor and the intricate communication between these cells and the tumor cells. **Figure 1** illustrates an overall view of the intricate workings of the TME and multifaceted interactions with tumor cells.

### 3.1 Immune evasion

The presence of tumor-associated immune cells within the TME poses a critical question that remains one of the foremost challenges in oncology: How do cancer cells evade the immune system? To address this question, it is essential to trace the progression from a physiologically anti-tumor microenvironment in the early stages of cancer development to an immune-suppressive microenvironment that promotes the survival and growth of cancer cells, a phenomenon commonly referred to as immune evasion (19).

**Figure 2:**
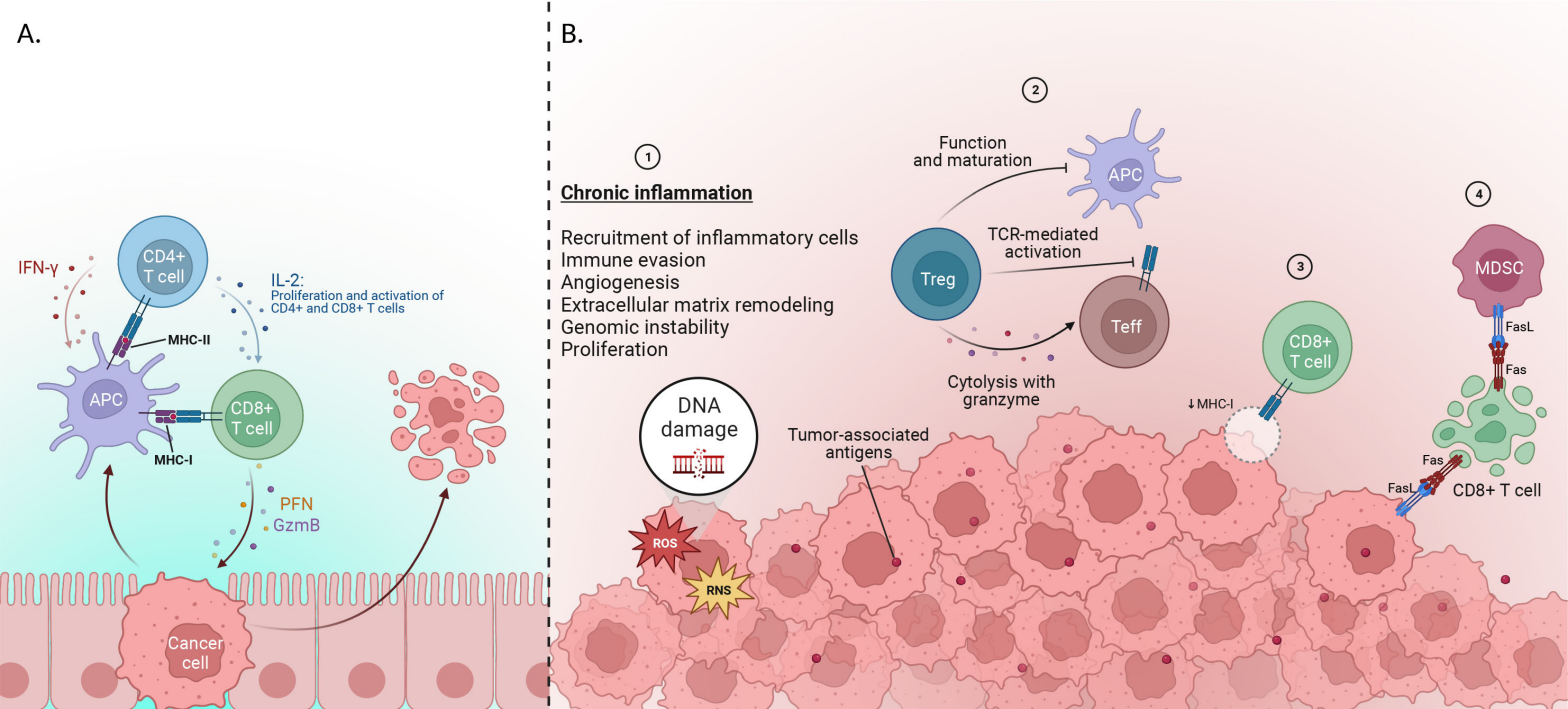
**(A)** Early stage of cancer development: Cytotoxic T lymphocytes (CTL), which are differentiated from CD8^+^ T-cells via IL-2, are able to destroy tumor cells by releasing perforin and granzymes. IL-2 plus IFN-γ is released by CD4^+^ cells after activation through MHC presented by APC cells. IFN-γ increases the expression of represented MHC by APCs to CD8^+^ T-cells and also enhances the differentiation of CD4^+^ cells into T helper-1(Th1). **(B)**Immune evasion mechanisms: 1) Genomic instability, immune evasion, angiogenesis, and metastatic dissemination are among the elements that lead to tumor progression. One factor that leads to the stimulation of cases is said to be chronic inflammation, which plays a vital role in the development of cancer. 2) various factors, including Tregs, suppress or activate effector immune cells. Inhibiting the immune system using suppressive molecules or different surface receptors, inhibiting the function of dendritic cells (DC), and releasing inflammatory cytokines are among the parts of Treg in this system. Also, inhibition of Teff activation in 2 ways by limiting TCR ligand binding and using granzyme and metabolic disorders are other functions of Tregs. 3) Furthermore, CTLs fail to recognize tumor cells, and this is due to the reduction of MHC-I presented by APCs to CD8^+^ T-cells. 4) Tumor cells use FAS ligand (FasL) proteins to suppress CTLs, leading to their apoptosis, and finally induce inhibition of immune response (Created with BioRender.com).

Typically, During the early stages of tumor development, anti-tumor immune cells can recognize and eliminate cancer cells. CD8^+^ T-cells are identified as the predominant anti-tumor cells. Once they are stimulated and activated by Antigen-presenting cells (APCs), CD8^+^ T-cells undergo differentiation into cytotoxic T lymphocytes (CTLs). These CTLs execute a potent anti-tumoral response by releasing granules containing perforin and granzyme, which directly destroy cancer cells (20–22). Moreover, activation of CD4^+^ T-cells results in the release of cytokines such as interferon-gamma (IFN-γ) (which increases expression of MHC-I, increases expression of major histocompatibility complex [MHC] molecules on APCs, promotes antigen presentation, promotes differentiation of CD4^+^ T-cells into a T helper-1 [Th1]) and interleukin-2 (IL-2) (which is a proliferative cytokine that activates other CD4 ^+^ and promotes CD8^+^ T-cell maturation into CTLs) (23) (**Figure 2**).

Nevertheless, many types of human tumors can suppress the immune system to enhance their survival (24, 25). Inflammation is thought to play a significant role in establishing an immune-suppressive TME. The significance of inflammation in cancer progression is not a recent hypothesis; Rudolph Virchow first proposed it in 1863. In his study, Virchow observed leukocytes surrounding cancer cells and suggested that cancer might arise from chronic inflammation (26). Inflammation is a host immune response that aims to limit infections or repair damaged tissue. While it can be beneficial for healing, persistent chronic inflammation can lead to cell and tissue alteration, increasing cancer risk (27).

In the present understanding of cancer, chronic inflammation is widely acknowledged as a crucial characteristic, with potential causes including persistent microbial infections, autoimmune disorders, and immune dysregulation. An illustrative example of this relationship is the increased risk of gastric cancer associated with chronic infection by Helicobacter pylori (28). Similarly, immune dysregulation observed in inflammatory bowel disease contributes to a higher incidence of colorectal cancer (29).

Long-term inflammation can also increase the risk of cancer development by releasing reactive oxygen species (ROS) and reactive nitrogen species (RNS), which can cause harm to the cellular components, including DNA. As a result, it may lead to genetic mutations that influence genes responsible for cell growth and division, triggering abnormal cell growth and tumor formation (30, 31).

As the tumor grows and the tumor TME undergoes alterations, excessive tumor-associated antigens are produced during tumorigenesis. Consequently, the immune system’s ability to prime new repertoires of T cells and direct them toward the tumor is affected, leading to changes in the effectiveness of tumor containment. In parallel, cancer cells and the TME employ mechanisms to suppress the anti-tumor function of the immune system, often through the recruitment of regulatory CD4^+^ T-cells (Tregs) (32). Tregs play a critical role in the priming, activation, and cytotoxicity of other effector immune cells, including CTLs, Th1 CD4^+^ T-cells, NK cells, macrophages, and neutrophils (33, 34).

Moreover, some tumor cells escape immune detection by decreasing the expression of specific antigen-presenting proteins (for example, MHC-I) at their surface, rendering them invisible to CTL (35, 36). But more often, tumors secrete proteins that inhibit CTLs to suppress immune responses. For example, Fas ligand (FasL) has been reported to be expressed by the tumor cells, MDSCs, and vascular endothelium in many human solid tumors (37, 38). Consequently, the presence of FasL triggers apoptosis in the CTLs (39).

Tumor cells employ various factors, such as Cyclooxygenase-2 (COX-2), Prostaglandin E2 (PGE2), IL-6, Granulocyte-macrophage colony-stimulating factor (GM-CSF), S100 proteins, and Snail, to recruit and expand MDSCs within the TME. MDSCs, in turn, exert immunosuppressive effects on T-cell activation through multiple mechanisms. These include the depletion of essential amino acids like L-arginine and cysteine from the TME, the production of ROS and peroxynitrite, and the downregulation of CD62L and T-cell activation. MDSCs also contribute to the induction of Tregs via the production of IL-10 and transforming growth factor-beta (TGF-β). Additionally, MDSC expansion and IL-10 production inhibit DC antigen presentation, further contributing to immune suppression within the TME (40).

The secretion of certain growth factors, including TGF-β and epidermal growth factor (EGF), can also lead to increased cell proliferation and decreased responsiveness to signals that usually trigger programmed cancer-cell death (41, 42). Over time, this unregulated growth can lead to the emergence of cancer (**Figure 2**).

### 3.2 Tumor-infiltrating lymphocyte

Amidst the diverse immune cell types that infiltrate cancerous tissues within the TIME, tumor-infiltrating lymphocytes are gaining recognition as a critical component due to their versatile functions and clinical potential (43). The formation of TILs from circulating lymphocytes is a complex process that begins with the migration of immune cells across the tumor endothelial barrier to reach the tumor site. However, the tumor endothelium is often disrupted and can directly suppress T cell function, impeding TIL infiltration. Notably, vessels carrying circulating lymphocytes are predominantly absent from the tumor core, instead localizing in the surrounding stroma and/or invasive margin, suggesting a directional aspect to TIL infiltration. These findings highlight the pivotal role of the tumor endothelium in regulating T-cell migration and infiltration and underscore the importance of the stroma in tumor development (44). Histopathological analyses of tumor samples have revealed distinct TIL distribution patterns across tumor types, with different immune cell types found in specific locations around and within the tumor. Interestingly, TIL distribution was observed to be non-random and organized in particular areas (45).

TILs generally encompass a diverse assortment of immune cell types, such as clusters of T-cells and B-cells (46, 47).

In the realm of cancer research, T-cells have received significant attention due to their prominence as the second most prevalent immune cell type found in human tumors across diverse cancer types. In the early stages of tumor development, if a sufficient number of immunogenic antigens are generated, naïve T cells undergo priming within the draining lymph nodes and/or bloodstream. This priming event is accompanied by their activation and subsequent migration to the tumor microenvironment (TME). Among the various types of T cells, the most widely recognized and extensively studied are the CD8^+^ T-cells (cytotoxic T-cells, or killer T-cells) and CD4^+^ T-cells (helper T-cells) (48).

**Figure 3:**
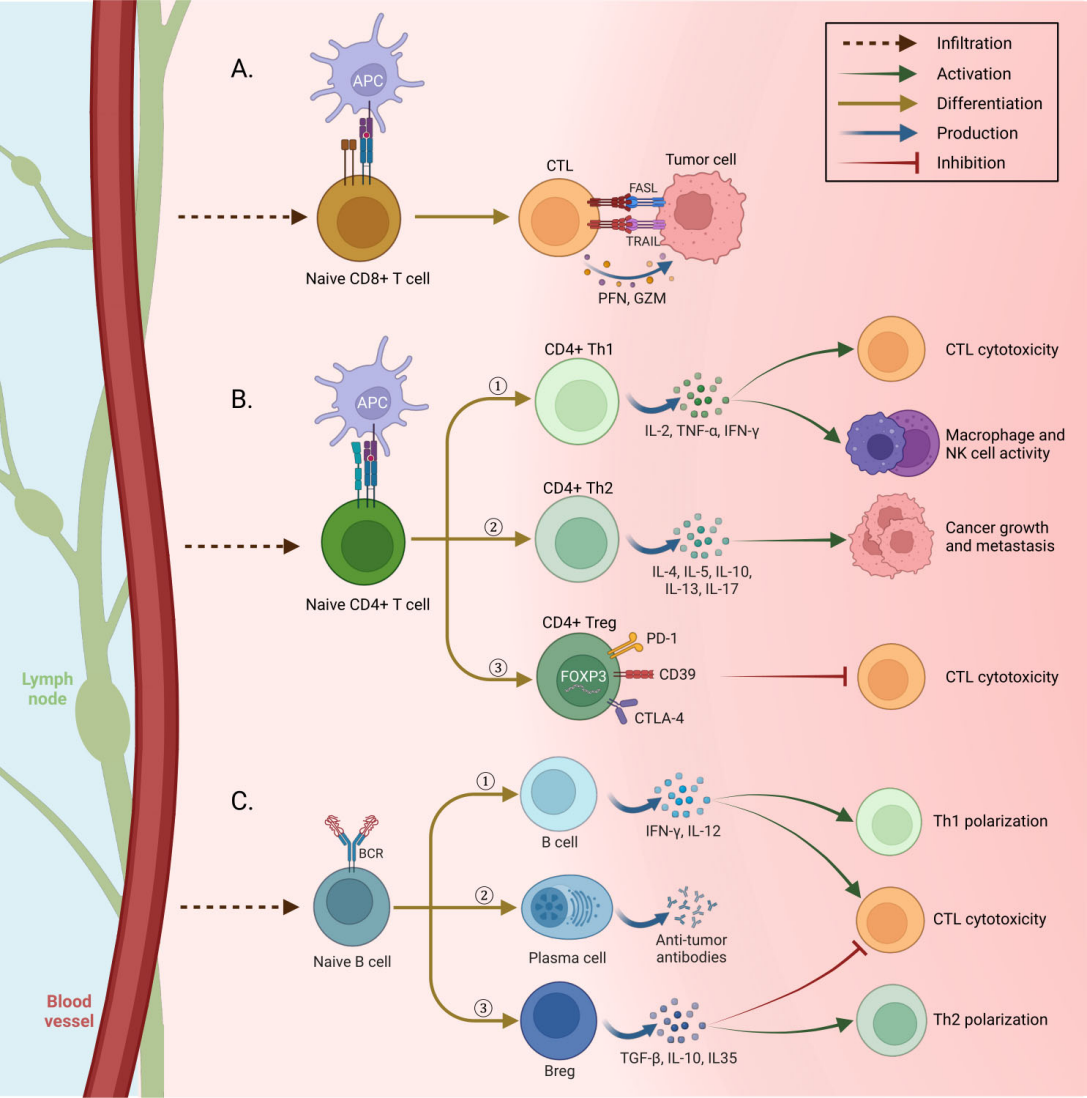
Immune cell differentiation in tissue. **(A)** Tumor cells are killed by two mechanisms when confronting cytotoxic T lymphocytes (CTL) activated by antigen-presenting cells (APC): first, cytolysis by releasing perforin and granzyme from CTLs. Second, the expression of death ligands FasL and TNF-related apoptosis-inducing ligands (TRAIL) on the surface of CTLs, whose interaction with the relevant death receptors leads to cancer cell apoptosis. **(B)** After penetrating the tissue and being activated by APCs, Naive CD4+ T cells differentiate into three different forms and have different functions: 1) Th1, secretion of IFN-g, TNF-a, and IL-2 to increase the toxicity and antitumor activity of CTLs, macrophages, and NK cells. 2) Th 2, by secreting different cytokines, promotes tumor cells to develop cancer. 3) Treg suppresses the cytotoxic activity of CTLs by using suppressor molecules. **(C)** After the penetration of naive B cells into the tissue, they are distinguished in three ways with specific functions: 1) Tumor-infiltrating B cells cause Th1 polarization and toxicity of cells by secreting IFN-γ and IL-12. 2) Plasma cells secrete tumor-specific antibodies and cause phagocytosis of tumor cells and stimulation of complement cascade. 3) Bregs, secretion of immune regulatory cytokines to Th2 polarization and inhibit the activity of CTLs (Created with BioRender.com).

Cytotoxic CD8^+^ T-cells (CTLs) of the adaptive immune system are the most potent effectors in the anti-cancer immune response. Activation of CTL is an antigen-specific process requiring the interaction of the T-cell receptor (TCR) complex with a processed tumor antigen–derived peptide bound to a MHC class I molecule presented by APCs or tumor cells (49). There are two mechanisms through which the CTLs carry out their function of combating cancer: granule exocytosis and the death ligand/death receptor system. The secretory granules comprising perforin and granzymes fuse with the plasma membrane and release their content, eliminating tumor cells. During death ligand/death receptor-mediated apoptosis, when CTLs are activated, FasL and TNF-related apoptosis-inducing ligands (TRAIL) are expressed on their surface. These ligands can kill susceptible cancer cells by interacting with their respective death receptors (50–52) (**Figure 3A**).

CD4^+^ T lymphocytes play a crucial role in regulating the immune response and, in turn, are subdivided into at least Th1, Th2, Th9, Th17, and T regulatory (Treg) groups based on cytokine profiles and immune functions (53, 54). CD4^+^ T-cells interact with antigens in the context of MHC class II. This interaction causes the secretion of cytokines from the CD4^+^ cells to regulate various immune cells (55).

CD4^+^ Th1 cells, characterized by their secretion of high levels of proinflammatory cytokines such as IL-2, tumor necrosis factor-α (TNF-α), and IFN-γ, play a crucial role in the anti-tumoral response. Their activities include promoting T-cell priming and activation, enhancing the cytotoxicity of CTLs, boosting the anti-tumoral activity of macrophages and NK cells, and increasing the presentation of tumor antigens (24, 56) (**Figure 3B**).

CD4^+^ Th2 cells exhibit the secretion of a distinctive array of cytokines, including IL-4, IL-5, IL-10, IL-13, and IL-17. However, it is noteworthy that not all of these cytokines confer beneficial effects in the context of cancer, as some have been implicated in promoting tumor progression within the Th2 subtype (57). Specifically, IL-4, IL-5, and IL-13 have been extensively demonstrated to contribute to cancer growth and metastasis (57) actively.

Tregs are identified by their expression of the transcription factor Forkhead box p3 (FOXP3) along with a specific combination of cell surface markers, including CD4^+^, CD25^+^, and CD127^low/−^. Among these markers, FOXP3 is considered the most reliable and specific cell marker for identifying Tregs. Its presence indicates the suppressive and regulatory functions associated with Tregs in immune responses (58). FOXP3^+^ Tregs are responsible for maintaining immune tolerance, which can prevent allergic and other kinds of autoimmune diseases as well as inhibit the anti-tumor immune responses (59, 60). Tregs exert their immunomodulatory effects by utilizing several suppressive molecules and mechanisms. They employ programmed death-1 (PD-1), cytotoxic T lymphocyte-associated antigen (CTLA-4), CD39, and diverse surface receptors to suppress immune responses. Tregs inhibit the function of DCs, which are crucial for initiating immune responses. Additionally, Tregs secrete anti-inflammatory cytokines such as IL-10, TGF-β, and IL-35, which dampen immune activation and promote an immunosuppressive environment. Furthermore, Tregs directly inhibit CTLs by eliciting cytolysis through the release of granzyme and by inducing metabolic disruptions (61, 62).

The balance between these CD4^+^ T-cell subsets is critical for maintaining immune homeostasis and preventing immune dysfunction in the TME (63).

B lymphocytes are the main cellular components of the humoral compartment of adaptive immunity (64). CD20 is a transmembrane protein found on the surface of B-cells. CD20 is a transmembrane protein found on the surface of B-cells. It is a marker of B-cells and is involved in B-cell development, differentiation, and B-cell receptor signaling (65).

CD20^+^ B-cells can exert both pro-tumor and anti-tumor effects on cancer growth (66). The balance between these opposing functions is influenced by various factors, including the interplay between B-cells and other immune cells, as well as the TME.

Evidence indicates that B-cells play several roles in promoting tumor progression (67). They can release IL-10, IL-35, and TGF-β that support Treg expansion and Th2 polarization while suppressing effector T-cell activity, which is potentialized by B-cell PD-L1 expression (68) (**Figure 3C**). Vascular endothelial growth factor (VEGF)-producing B-cells may also promote tumor progression through neoangiogenesis (67, 69).

B-cells can also contribute to suppressing tumor progression through various mechanisms. B-cells have the ability to present tumor-derived antigens to T-cells, facilitating their activation and the subsequent immune response against tumors. Furthermore, B-cells secrete cytokines such as IFN-γ and IL-12, which support the polarization of CD4^+^ T-cells towards a Th1 phenotype and enhance the cytotoxicity of CD8^+^ T-cells. Tumor-specific antibodies produced by plasma cells, a differentiated form of B-cells, play a crucial role in tumor immunity. These antibodies can trigger the complement cascade, leading to the destruction of tumor cells. They can also mediate the phagocytosis of tumor cells by immune cells and facilitate antibody-dependent cell cytotoxicity (ADCC) mediated by natural killer (NK) cells.

Moreover, activated B-cells can directly eliminate tumor cells by secretion molecules such as TNF-related apoptosis-inducing ligand (TRAIL) and granzyme B. These effector molecules induce apoptosis in tumor cells, contributing to their elimination (67, 70, 71).

## 4 Clinical significance of tumor-infiltrating lymphocytes in gastrointestinal tract cancers

More than two centuries ago, Rosenberg et al. (72) conducted a study that indisputably proved that TILs can enhance the condition of more than 50% of colon adenocarcinoma-bearing mice with hepatic or pulmonary metastasis when combined with cyclophosphamide and IL-2 (72, 73). Despite this conclusive discovery, the use of TILs in cancer treatment is still not prevalent in clinical settings. This unequivocally emphasizes the dire need for further research and adoption of TILs as a potential solution in GI cancer treatment. Here, we present a comprehensive overview of recent scientific advancements in the role of TILs in Esophageal cancer (**Table 1**), Pancreatic cancer (**Table 2**), Gastric cancer (**Table 3**), Colorectal cancer (**Table 4**), and Hepatocellular cancer (**Table 5**). Specifically, we summarize these developments in the context of cancer prognosis, treatment strategies, and the associated challenges and opportunities for further advancement.

**Table 1:** Esophageal cancer.

First author	Cancer	Tumor stage	No. of patients	Markers	Result (s)
Zhou et al. (2020) (74)	nCRT for EC	II-IVa	138	IDO1/PD-L1/CD8	Elevated post-nCRT CD8+ density associated with improved OS and recurrence-free survival/The reduced IDO1 expression and increased CD8+ density following CRT were linked to improved survival outcomes.
Kovaleva et al. (2020) (75)	ESCC	I–IV	48	Macrophages/CD3/CD8/CD68/CD163/CD206/PU.1/iNOS/FOXP3/PD-L1	FOXP3^+^ cells connected to good prognosis in the analysis of OS/no notable correlations between CD3+ and CD8+ cells and the tumor’s clinical characteristics.
Conroy et al. (2021) (76)	SCC/EAC	I–IV	95	CD3/CD8/CD45RO/CD56/CD68/CD69/CD107/IFN-γ/IL-4/IL-10/IL-17/TGF-β/FOXP3	Higher expression of CD45RO^+^, CD8^+^, and CD3^+^ within SCC stroma than EAC. Pro- and anti-inflammatory profile of EC expression of IL-17 and TGF-β in the stroma of both EAC and SCC higher expression of IFN-γ and FoxP3 in SCC. Higher IL-10 expression in EAC. CD3^+^, CD8^+,^ and CD45RO^+^ are not associated with OS.
Haddad et al. (2022) (77)	EAC	I-II-III	43	CD3/CD4/CD8/CD20/ CD45RO/CD68/ CD163/FOXP3	CD8^+^ independent prognostic factor for OS. High levels of CD3^+^, CD4^+^, CD8, and CD45R0 TILs correlate with better DFS. Higher level of TILs (CD3^+^, CD4^+^, CD8^+^, and CD45R0^+^) associated with tumor response after neoadjuvant chemotherapy
Soeratram et al. (2022) (78)	EAC	I–IV	188	PD-L1/FOXP3/CD8/TAICs	The high density of CD8^+^, FOXP3^+^, PD-1^+^, and TAICs is associated with a better response. Increased density of TAICs in pretreatment biopsies related to response to nCRT. High PD-1+TAIC density associated with worse OS
Nomoto et al. (2022) (79)	EC	I–IV	433	PD-1/PD-L1/CD57/CD8/CD27	↑ PD-1 expression on TILs is associated with high overall mortality, ↑PD-1 and PD-L1 expression is associated with worse OS
Noma et al. (2023) (80)	EC	I–IV	300	CD3/CD8	IS as an independent prognostic factor positively associated with better OS
Hui Wang et al. (2023) (81)	ESCC	I–IV	118	CD4/CD8/FOXP3/IL-10/IgG4	CD4^+^ is associated with better prognosis, higher density of CD4^+^ + CD8^+^ correlates with better survival, IgG4 positive B-cells in serum are associated with poor prognosis

ALC, Absolute lymphocyte count; CSS, Cancer-specific survival; CT, Tumor center; DFS, Disease-free survival; EAC, Esophageal adenocarcinoma; EC, Esophageal cancer; ESCC, Esophageal squamous cell carcinoma; IDO1, Indoleamine 2;3-dioxygenase-1; IFN-γ, Interferon-gamma; IgG4, Immunoglobulin G4; IL-10, Interleukin-10; iNOS, Inducible nitric oxide synthase. MAGE-C2, Melanoma-associated Ag-C2; nCRT, Neoadjuvant chemoradiation therapy; OS, Overall survival; PD-1, Programmed Cell Death Protein 1; PDL-1, Programmed Cell Death Ligand 1; PFS, Progression-free survival; PSCCE, Primary small cell carcinoma of the esophagus; sTIL, Stromal tumor-infiltrating lymphocytes; TAICs, Tumor-associated immune cells; TC, Tissue microarray; TGF-β, Transforming growth factor-β; TP, Tumor periphery.

**Table 2:** Pancreatic cancer.

First author	Year	Cancer	Tumor stage	No. of patients	Markers	Result(s)
Lianyuan et al. (82)	2018	PDAC	I–IV	155	TIL(CD45)	Low stromal TIL associated with lower OS and higher liver metastasis
Zhang et al. (83)	2018	PDAC	I–IV	143	CD8/CD4	CD8+ T-cell independently contributes as a favorable factor for OS. CD4^+^ T-cells had a controversial role in prognosis.
Tahkola et al. (84)	2019	PDAC	I–III	79	CD3/CD8	Higher ICS is associated with better OS
Delayre et al. (85)	2020	PAC	I–IV	43	CD4/CD8/CD3/CD45/FOXP3	High CD3^+^ favorable pathological characteristic. High CD4^+^/CD3^+^ ratio linked to reduced survival. Low FOXP3^+^/CD8^+^ ratio associated with longer DFS
Fraune (86)	2020	PDAC adenocarcinomas of the ampulla of Vater and acinar cell carcinomas	II	597	MMR proteins MLH1, PMS2, MSH2, or MSH6/ CD8	MMR deficiency is associated with higher CD8+ infiltration
Başoğlu et al. (87)	2022	PDAC	I–III	81	CD103/CD204/ PDL-1	intra-tumoral CD103 expressing CD8^+^ T-cells are associated with OS and DFS. No correlation between PDL-1 and survival

DFS, Disease-free survival; ICS, Immune cell score; MMR, Mismatch repair; OS, Overall survival; PDAC, Pancreatic ductal adenocarcinoma; PDL-1, Programmed Cell Death Ligand 1, TILs; Tumor-infiltrating lymphocytes

**Table 3:** Gastric cancer.

First authors	Year	Cancer	Tumor stage	No. of patients	Markers	Result(s)
Kim et al. (88)	2017	GC	I-IV	153	CD3/CD8	PDL-1 positive and high IS are correlated with better prognosis
Jiang et al. (89)	2019	GC	I-IV	879	CD3/CD8/CD45RO/CD66b	High IS is associated with better OS and DFS
Zhang et al. (90)	2019	GC	I-III	833	TIL	High TIL correlates with better OS
Kemi et al. (91)	2020	GC	I-IV	741	CD3, CD8, and KM grade	Both IS and KMgrades are prognostic factors in GC.
Yun et al. (92)	2021	GC	II/III	389	CD3, CD8, and FOXP3	High IS associated with better DFS and OS in both MSS/MSI-low and MSI-high group
Zou et al. (93)	2021	GC	II-III	101	CD3 and CD8	IS positively correlated with better OS and DFS
Ni et al. (94)	2021	GC	I-IV	584	CD20	High CD20^+^ B cell associated with better OS and DFS
Yan et al. (95)	2021	GC	I/III	273	DC, Mast cell, CD4, CD8, Th17, CD56, Ba, Bm	Low IS group had longer DFS and OS. Low IS with more TIL had better response to adjuvant chemotherapy

DFS, Disease-free survival; GC, Gastric cancer; IS, Immunoscore; KM grade, Klintrup–Mäkinen grade; MSI, Microsatellite instability; MSS, Microsatellite Stability; OS, Overall survival; PDL-1, Programmed Cell Death Ligand 1; Th17, T-helper 17; TILs, Tumor-infiltrating lymphocytes.

**Table 4:** Colorectal cancer.

First author	Year	Cancer	Tumor stage	No. of patients	Markers	Result(s)
Williams et al. (96)	2019	CRC	II/III	1256	TIL	Despite the MSI status, TIL-low status showed poor DFS
Chalabi et al. (97)	2020	CRC	I-III	40	CD8, PD-1	CD8^+^ PD-1^+^ T-cell infiltration predicted pathologic response neoadjuvant chemotherapy in pMMR tumors (27% of patients)
Pagès et al. (98)	2020	CRC	III	1322	CD3 and CD8 (_CT+ IM_)	IS was notably independently linked to DFS when considering adjustments for sex, histological grade, T/N stage, and MSI.
Mlecnik et al. (99)	2020	CRC	III	763	CD3 and CD8 (_CT+ IM_)	IS as a predictor for CRC patients’ survival. RFS was 56.9%, 65.9%, and 76.4% in patients with low, intermediate, and high IS, respectively
Reichling et al. (100)	2020	CRC	III	1018	CD3, CD8 (_CT+ IM_)	Higher CD3^+^_CT_, CD3^+^_IM_, and CD8^+^_CT_ densities were significantly associated with a more prolonged RFS.
Zou et al. (101)	2021	CRC	I-IV	Two cohorts: 282 and 335	CD8 MeTIL	A lower CD8+ MeTIL score (abundance of CD8+ TILs) was linked to MSI-H tumors and predicted enhanced survival in cohorts of CRC patients.
Marie et al. (102)	2021	CRC	IV	24	PDL-1, CD20 and CD73	A B-cell transcriptome signature and B-cell density increase were present in post-treatment samples from patients with prolonged RFS.
Johnson et al. (103)	2022	CRC	IV	29	CD8	Higher initial density of CD8+ TIL was linked to an increased probability of deriving benefits from immunotherapy.
Kuang et al. (104)	2022	CRC	IV	30	CD8	Higher initial density of CD8+ TIL was linked to an increased probability of deriving benefits from immunotherapy.
Elomaa et al. (105)	2022	CRC	I-IV	983	CD3 and CD8	A high T-cell proximity score was associated with longer CSS
Xin et al. (106)	2023	CRC	IV	111	CD4, CD8, and PDL-1	CD8^+^ infiltration in primary tumors independently served as a predictive factor for OS.

CD8+ MeTIL, DNA methylation signature for CD8+ TILs; CRC, colorectal cancer; CSS, cancer-specific surviva; CT, Tumor center; DFS, Disease-free survival; IM, Invasive margin; IS, Immunoscore; MSI, Microsatellite instability; OS, Overall survival; pMMR, Proficient mismatch repair, RFS, Relapse-free survival; T/N stage, Tumor/Nodal stage; TIL, Tumor-infiltrating lymphocytes.

**Table 5:** Hepatocellular cancer.

Author	Year	Cancer	Tumor stage	No. of patients	Markers	Result(s)
Wang et al. (107)	2019	HCC	I-IV	40	TOX on CD8 T-cell	Elevated levels of TOX in peripheral CD8+ T-cells are associated with diminished effectiveness of anti-PD1 treatments and poorer prognosis
Kim et al. (108)	2020	HCC	I-IV	79	4-1BB	Increased 4-1BB (pos) cell expression on CD8+ TILs is linked to tumor reactivity, T-cell activation, and better prognosis.
Gao et al. (109)	2021	HCC	I-IV	Two phases: 315 and 343	TIL	Higher TILs are associated with OS and DFS
Stulpinas et al. (110)	2023	HCC	I-IV	106	CD8 in tumoral and non-tumoral region	High CD8^+^ density in tumor edge and low CD8^+^ density within non-tumoral is associated with OS. Higher CD8^+^ density in the epithelial stroma is associated with higher RFS.
Kuwano et al. (111)	2023	HCC	I-IV	39	CD8 T-cell	High CD8^+^ TILs group had longer PFS Atezolizumab plus bevacizumab group.

DFS, Disease-free survival; HCC, Hepatocellular carcinoma; OS, Overall survival; PD-1, Programmed Cell Death Protein 1; TILs, Tumor-infiltrating lymphocyte; TOX, Thymocyte selection-associated HMG BOX.

### 4.1 Esophageal cancer

Based on recent advances in cancer treatment, immunotherapy is crucial in the therapeutic strategy for various cancers, including esophageal cancer (EC) (112). Despite the advances, immunotherapy is not a highly effective treatment for EC patients as the overall response rate is less than 30%, and despite being initially treated with immunotherapy, most patients tend to develop acquired resistance over time (113). Due to the heterogenicity of the TIME in EC, the cause of resistance to immune therapy is still unclear (114). TILs, as extrinsic factors, can participate in cancer development and response to immunotherapy (79). The presence and number of TILs in the tumor environment might predict patients’ prognosis and cancer outcome (115).

Haddad R et al. (77) explored the association between neoadjuvant treatment, TILs, and survival in patients with EC who underwent esophagectomy. Their investigation of Forty-three specimens of EC showed higher abundant of CD8^+^, CD4^+^, CD3^+^, and CD45R0^+^ cells was positively correlated with praising pathological response to neoadjuvant chemotherapy, disease-free survival (DFS), and overall survival (OS) (77). Findings from the multivariate analysis also showed that CD8^+^ in the stroma is an independent factor in the prognosis of EC patients (77) (**Table 1**). In another study, the expressions of CD8^+^, CD4^+^, FOXP3^+^, Immunoglobulin G4 (IgG4), and IL-10 and clinical information of 118 patients with esophageal squamous cell carcinoma (ESCC) were assessed with hematoxylin and eosin (H&E), immunohistochemistry (IHC) staining and multi-color Immunofluorescence (81). The higher expression of CD4^+^ (OS, hazard ratio [HR]= 0.395, *P-value*= 0.007) and CD4^+^ plus CD8^+^ (OS, HR= 0.478, *P-value*= 0.013) was positively correlated with better prognosis and survival, and also CD4^+^ (OS, HR= 0.317, *P-value*= 0.008) was an independent protective factor for ESCC patients. Higher IgG4 in serum was shown to be associated with poorer prognosis. Additionally, CD4^+^ regulates IgG4-positive B lymphocytes in TME by producing IL-10 (81).

**Figure 4:**
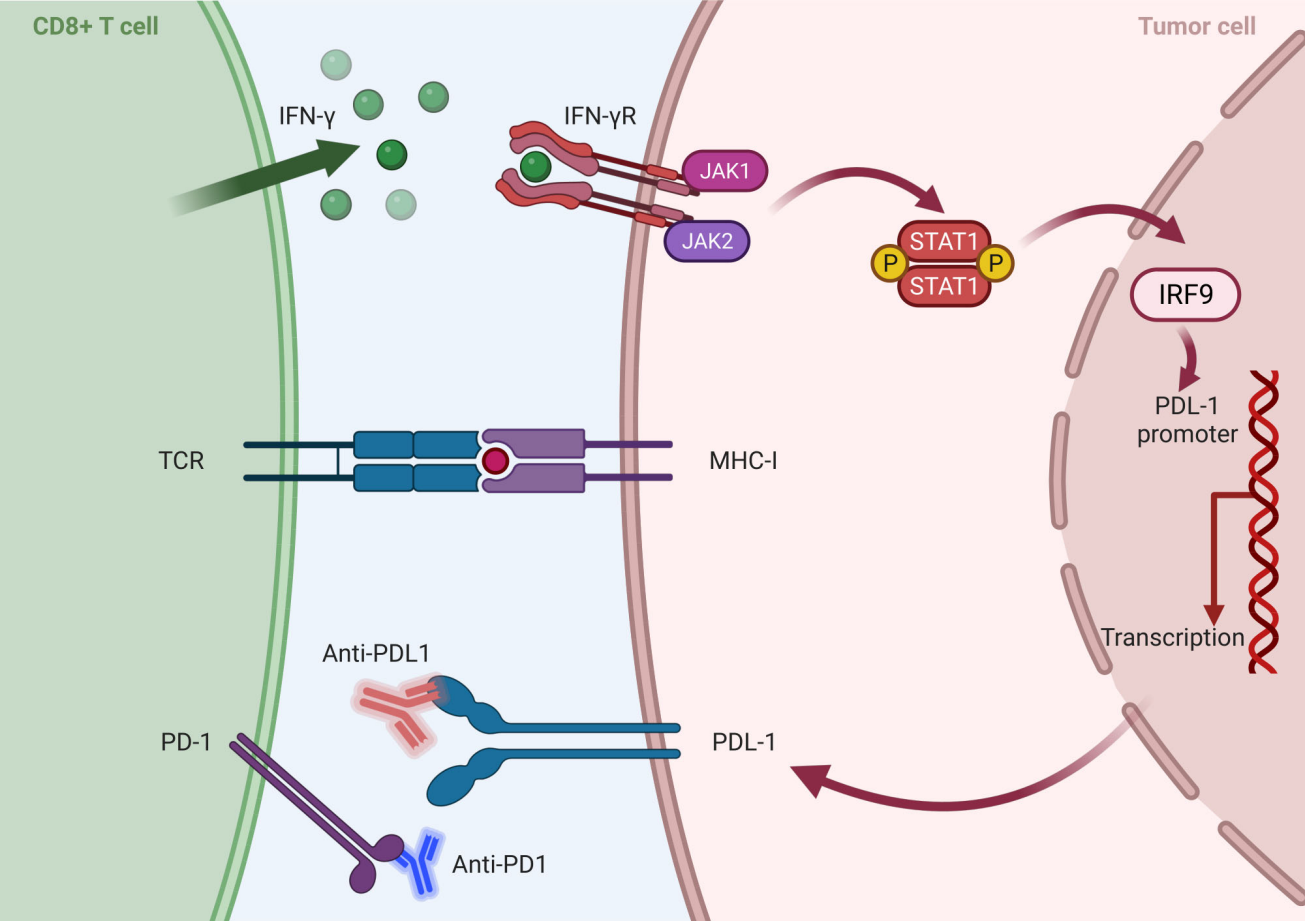
Mechanism of inhibition of PD1/PDL1 axis by tumor cells: After activation of CD 8^+^ T-Cell by MHC-I, IFN is secreted and activates the transcription factor IRF in the nucleus and, finally, the expression of PDL 1 in tumor cells. On the other hand, TCR signaling leads to an increase in the PD1 expression on the surface of T cells and the activation of the PD 1/PDL 1 axis, and the interactions between these two ultimately reduce the antitumor effects of T cells. Anti-PD1/PDL1 antibodies, as an effective treatment method, block the PD1/PDL1 function and increase immune cells’ antitumor activity (Created with BioRender.com).

Within developing and inspiring immunotherapies, immune checkpoint inhibitors (ICBs) targeting the programmed cell death 1/programmed cell death ligand 1 (PD1/PDL1) axis have garnered notable recognition, including the prestigious Nobel Prize in 2018 (116). These inhibitors have been granted approval for application across various solid tumors. Upon encountering the MHCs, T-cells release IFN-γ, thereby augmenting the efficiency of tumor eradication. In response to IFN-γ released by CD8+ T-cells, the expression of PDL1 is upregulated on tumor cells. Concurrently, TCR signaling triggers the upregulation of PD1 on the surface of T-cells. The interaction between PD1 and PDL1 imposes negative regulatory effects, attenuating the anti-tumor function of T-cells (117, 118) (**Figure 4**). T-cells previously rendered inactive can be revitalized by targeting the PD1/PDL1 interaction through immunotherapy, reinstating their potent anti-tumor capabilities. The PD1/PDL1 blockade is emerging as a hopeful treatment option for cancer, showing impressive antitumor reactions while causing only minor side effects (119).

Despite recognizing anti-PD1/PDL1 therapy as a significant breakthrough, clinical data have revealed limited response rates. Studies have shown that a substantial proportion of patients exhibit primary resistance, failing to respond to PD1/PDL1 blockade, while some other initial responders eventually develop acquired resistance (120). The association between the expression of PDL1 and PD1 and its impact on cancer prognosis continues to be a subject of ongoing scientific debate (121). Furthermore, the underlying mechanisms contributing to primary and acquired resistance to PD1/PDL1 therapy remain largely elusive, presenting a significant challenge in the field (122). Researchers are actively investigating the intricate association between PD1/PDL1 expression and TIL composition to unravel the underlying complexities and understand these interactions more deeply. Advancements in this area promise to improve the efficacy of PD1/PDL1-targeted therapies and ultimately optimize clinical outcomes (121).

In a large cohort study involving 433 patients who underwent curative resection for EC, the expression of PD-1 on TILs and cancer cells was meticulously evaluated (79). The study assessed the relationship between PD-1 expression and OS in patients with and without preoperative treatment. Notably, high PD-1 expression on TILs was found to be associated with poorer OS, specifically in individuals who did not receive preoperative therapies. Conversely, no significant difference in OS was observed in patients who underwent preoperative treatments. Furthermore, the researchers implemented a classification system that categorized patients into three distinct groups based on the expression levels of PD-1 and PD-L1. Group 1 encompassed individuals with high expression of both PD-1 and PD-L1, Group 2 consisted of patients with high PD-1 expression but low PD-L1 expression (or vice versa), and Group 3 consisted of individuals with low expression of both PD-1 and PD-L1. Notably, a significant disparity in OS was observed among these three groups, as evidenced by a log-rank *P-value* of 0.0017. Specifically, the 3-year OS rates were 58% in Group 1, 65% in Group 2, and 74% in Group 3 (79).

Additionally, Soeratram et al. (78) explored immune landscape patterns in the TME before and after neoadjuvant chemoradiation (nCRT). They were applying a comprehensive image analysis of digital image whole slides.; they revealed that the high mean density of combined CD8^+^, FOXP3^+^, and PD-1^+^ TILs in tumor epithelium (in tumor nest contact with tumor cells; distance < 20μm) and stroma (in tumor stroma not contact with tumor cells; distance > 20μm) of biopsies was associated with the better histopathological response (tumor regression grade) in 188 post‐nCRT resected specimens, and only CD8^+^ was associated with outcome (78). With the aim of evaluating the changes in immune markers after nCRT and the prognostic significance in esophageal squamous cell carcinoma (ESCC), Zhou et al. (74) analyzed indoleamine 2,3-dioxygenase 1 (IDO1), CD8^+^, and PD-L1 expression in 138 patients with ESCC who underwent nCRT and esophagectomy without achieving complete pathologic response were included for analysis. They demonstrated that the expression levels of IDO1[an immune inhibitor that suppresses T-cell function (123, 124)], PD-L1, and CD8^+^ density increased significantly after nCRT (*P-value* < 0.01 for all). Patients with high IDO1 expression after nCRT had poorer OS (*P-value* = 0.001). High post-CRT CD8^+^ density was significantly correlated with more favorable OS (*P-value =* 0.01) and relapse-free survival (RFS) (*P-value =* 0.008). However, neither pre- nor post-CRT PD-L1 expression was an independent prognostic factor for survival (74).

Interestingly, infiltration of TILs can be divergent in different types of EC, in which a study in 2021 by Conroy et al. showed that expression of CD8^+^ and, CD45RO^+^, and FOXP3^+^ are higher in squamous cell carcinoma (SCC) in comparison to esophageal adenocarcinoma (EAC) (76). However, this study also claims that there is no association between the expression of CD3^+^, CD8^+,^ and CD45RO^+^ in the tumoral and stromal regions of EAC and SCC with OS (76). Despite all, a recent meta-analysis of 30 articles comprising 5,122 patients for the role of TILs in the prognosis of EC patients revealed that increased levels of generalized TILs (HR= 0.67, 95% CI= 0.47–0.95, *P-value* = 0.02), CD8^+^[HR= 0.68, 95% confidence interval (95% CI)= 0.60–0.78, *P-value <*0.001], and CD4^+^(HR= 0.70, 95% CI= 0.57–0.85, *P-value <*0.001) were associated with better OS and is not correlated with DFS. However, high levels of CD3^+^ and FOXP3^+^ were not correlated with OS and DFS of EC patients (125).

### 4.2 Pancreatic cancer

Pancreatic cancer (PC) is one of the poor prognosis cancers with a survival rate of 5 years below 5% (126). Poor perfusion environment, which leads to a reduction in the distribution of treatment agents and immune cells into the tumor, and also low TILs infiltration in the core of the tumor, which is called “cold tumor,” are the main reasons for treatment challenges and progression of PC cancer (127, 128)

Tahkola et al. (84) assessed the density of CD8^+^ and CD3^+^ in tumor area and invasive margin (IM) in 79 pancreatic ductal adenocarcinomas (PDAC) patients after surgery by digital image analyses and introduced immune cell score (ICS) (84). The patients were divided into three groups with a range of ICS 0 (low CD3^+^ and CD8^+^ densities) to ICS 4 (high CD3^+^ and CD8^+^ densities) in both regions. The results revealed that higher ICS is associated with better OS and prognosis (84). They also compared two techniques for evaluating TILs in tumoral tissues. They found that the correlation of TIL density in the whole-section technique with survival is higher than in the hotspot technique (84) (**Table 2**).

The study evaluated the density of TILs in 155 surgically resected PDAC tissues using H&E staining. The tissues were divided into two groups based on their TIL density: stromal TIL-positive and stromal TIL-negative. Results showed that stromal TIL-negative status was an independent predictor of both worse OS (HR=2.80, 95% CI= 1.75-4.48, *P-value <*0.01) and liver metastasis (HR=2.7, 95% CI= 1.80-4.06, *P-value <*0.01) (82). Interestingly, this study claimed that TILs can hinder cancer progression through the secretion of TNF-alpha on tumoral cells (82). Previous studies propose that TNF-alpha can induce apoptosis in cancer cells, and its role in different cancers, such as pancreatic and colorectal cancer, has been proved (129–131).

Zhang and colleagues (83) reported that the location and distribution of TILs in the TME can impact the prognosis of patients with PDAC. They conducted IHC staining on 143 PDAC samples to evaluate TILs in the intraepithelial (lymphocytes in direct contact with tumor cells) and intratumoral (lymphocytes within the tumor tissue) regions. The study found that CD8^+^ T-cell intraepithelial attack was an independent favorable prognostic factor for OS and negatively correlated with vascular invasion. Conversely, high intratumoral CD8^+^ T-cell infiltration without CD8^+^ T-cell intraepithelial attack was a poor prognostic factor, accompanied by T-stage progression. The study highlights the potential importance of intraepithelial immune responses in developing and treating PDAC (83).

Delayre et al. (85) used tissue microarray from 43 patients with left-sided (body and tail) PC specimens that went through IHC of TILs (CD8^+^, CD 45^+^, CD3^+^, CD4^+^, FOXP3^+^), CAFs (vimentin, α-smooth muscle actin αSMA), and functional markers (PD-L1 and Ki-67) to examine their association with DFS and OS using computer-assisted quantitative analysis (85). Results proposed that a high CD4^+^/CD3^+^ lymphocyte ratio, along with a low αSMA/vimentin ratio, is correlated with poorer survival. Further, a high FOXP3^+^/CD8^+^ ratio was also associated with poorly differentiated tumors (85).

CD8^+^ memory T-cells (like non-circulating tissue-resident memory cells) stay long in tumoral tissue and play a crucial role in immune suppression (132). The members of tissue-resident memory T-cells induce their anti-cancer effects through overexpression of E-cadherin and improve the cytotoxicity of T-cells (133). A study on 81 operated PDAC patients was done to investigate the prognostic role of tissue-resident memory T-cell and TME features (87). Samples from intra-tumoral and peritumoral areas for evaluating tissue-resident memory cells, TILs, CD204^+^ macrophages, tumor stroma, and PDL1^+^ have been collected and underwent the staining process. Higher expression of intra-tumoral tissue-resident memory cell counts was associated with better survival (*P-value* = 0.84) in PDAC patients (87). Decreased survival was observed in tumors with increased CD204^+^ tumor-associated macrophages, which were immunosuppressive elements of the microenvironment (*P-value* = 0.29). Also, there was no correlation between the expression of PDL-1^+^ and survival in 81 operated PDAC tissues (87).

A systematic review and meta-analysis, which included 39 investigations on PDAC, was done by Orhan et al. (134) in 2020. They revealed that upregulation of CD8^+^ and CD3^+^ are associated with better OS (HR= 0.58, 95% CI= 0.50-0.68) and (HR= 0.58, 95% CI= 0.50-0.68) respectively, but increased levels of FOXP3^+^ is correlates with worse OS (HR= 1.48, 95% CI= 1.20-1.83). Interestingly, the role of CD4^+^ in OS is ambiguous (134). Also, there was no significant difference in the location of immune cell infiltration in the tumoral tissue (134).

**Figure 5:**
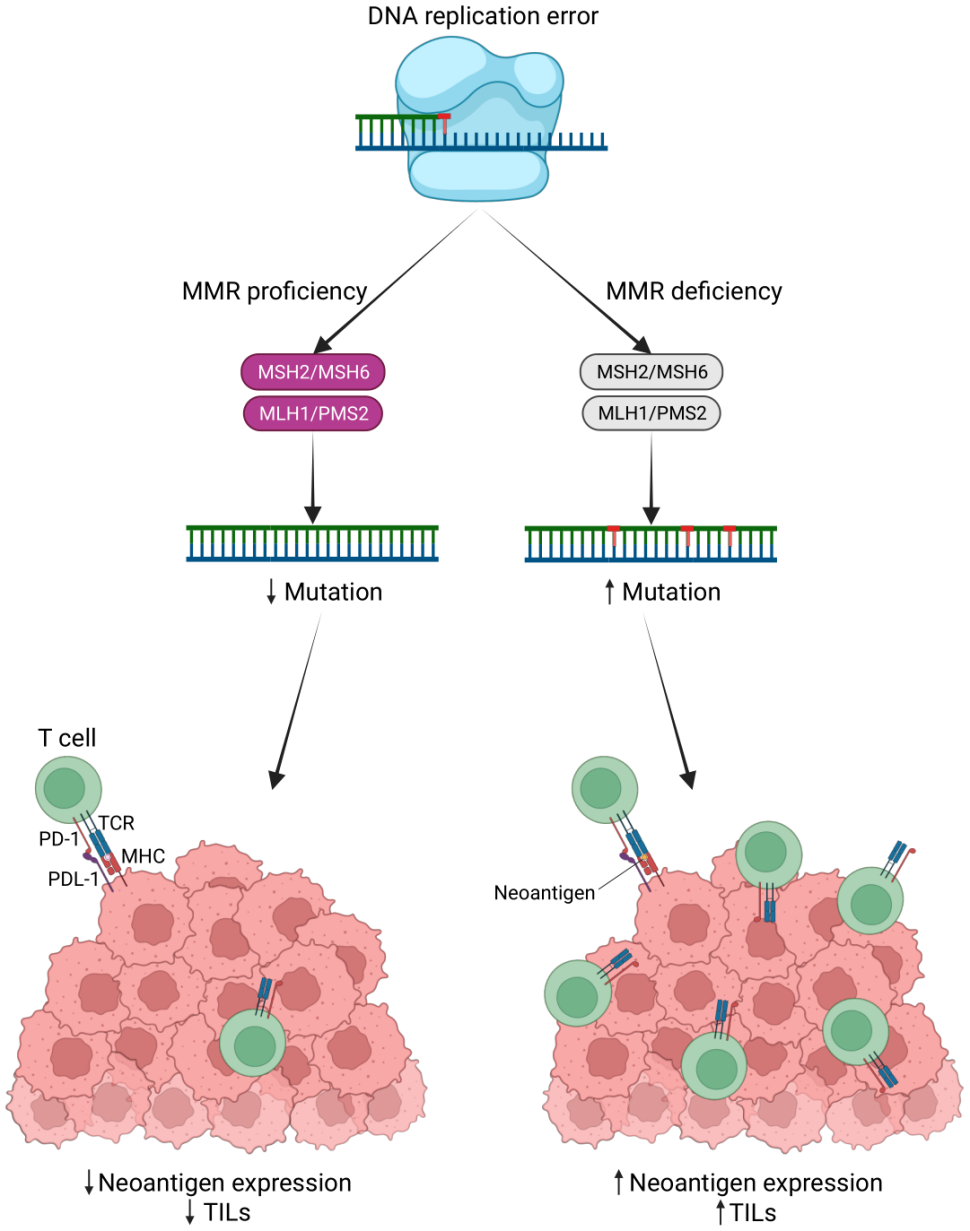
MSI status leads to a potent immune response. Neoantigens are produced by tumor cells due to genomic mutations, such as mutations in the DNA mismatch repair system (MMR) that lead to microsatellite instability (MSI). These antigens interact with T cell receptors (TCR) to increase the production of TILs. TILs recognize these antigens and induce a robust immune response (Created with BioRender.com).

Microsatellites are short, repetitive DNA sequences that are susceptible to errors when DNA replicates. Microsatellite instability (MSI) arises from DNA mismatch repair system errors, causing the accumulation of mutations in microsatellites. There are three MSI categories: MSI-high (MSI-H), MSI-low (MSI-L), and microsatellite stable (MSS) (135, 136). Notably, MSI-H/deficient MMR (dMMR) tumors have a 20-fold higher mutation rate compared to MSS tumors, and over 80% of MSI-High tumors exhibit a high tumor mutation burden (TMB) exceeding 20 mutations per megabase (Mb). TMB positively correlates with the number of neoantigens in various cancer types (137). TMB is recognized as a predictive marker for the response to cancer immunotherapy (138, 139). High TMB can generate numerous neoantigens that stimulate an anti-tumor immune response, potentially leading to improved responses to immunotherapy (140). The elevated mutational burden and frequent frameshift mutations in MSI-H/dMMR tumors result in the production of numerous neoantigens recognized by the immune system, which can trigger lymphocytic infiltrates (135, 141) (**Figure 5**).

To study the heterogeneity of MSI status in PC, a tissue microarray containing 597 tumors was examined through IHC using MutL protein homolog 1 (MLH1), postmeiotic segregation increased-2 (PMS2), MutS homolog 2 (MSH2), and MSH6 antibodies to detect MMR proteins and automated digital image analysis of CD8^+^. The results demonstrated markedly higher CD8 ^+^ in tumors “with” than “without” MMR deficiency (*P-value* < 0.0001), suggesting a role of MSI in the immune response. The significantly higher CD8 density in MMR deficient compared with MMR intact PCs may thus provide an additional hint towards a potential utility of ICI in the PCs (86). In a study involving 108 patients with PDAC, Tahkola et al. (142) assessed the Immune Cell Score (ICS) by quantifying the number of immune cells (CD3+ and CD8+) within the tumor core and invasive margin. The evaluation involved the utilization of tissue microarrays, IHC, and digital analysis, along with the application of MLH1 immunostaining to identify tumor tissues with MSI. The study found a significant correlation between a high ICS and improved disease-specific survival (DSS) and OS in PDAC patients. Notably, there was no observed connection between MSI status and ICS or the survival outcomes of individuals with PC (142). Still, more efforts must be made to investigate the association of MSI and TIL infiltration in patients’ prognosis and clinical characterization.

### 4.3 Gastric cancer

Advanced gastric cancer (GC) continues to pose a significant challenge, with a relatively low median survival of approximately 12-15 months (143, 144). It has become evident that relying solely on the TNM staging system is insufficient for accurately predicting prognosis and determining the appropriate benefits of adjuvant chemotherapy for patients with stage II and III GC following surgical intervention. As a result, researchers are actively investigating additional factors that can complement the TNM staging system in order to improve prognostic accuracy and guide treatment decisions for these patients.

Early in 1922, McCarty was the first one who brought up the concept of TILs, and considered the infiltration of lymphocytes into tumor tissue as an antitumor activity of the immune system and suspected that this could be a positive factor for the post-operative life of GC patients (145). Considering the role of the immune system in cancer development, in recent years, many efforts have been made to develop a comprehensive immune system to provide more accurate prognoses for GC patients.

A study investigated an Immunoscore system that assessed the density of CD3^+^, CD8^+^, and PD-L1 in the epithelial and stromal compartments of the tumor center (CT) and IM in 153 patients with MSI-H GC. Combining the analysis of PD-L1 expression and Immunoscore, the patients were classified into four distinct subgroups, demonstrating a significant difference in OS. The PD-L1 (+)/Immunoscore Low group exhibited the worst prognosis, while the PD-L1 (+)/Immunoscore High group showed the best prognosis. Multivariate analysis identified the combined status of PD-L1 expression and Immunoscore as an independent and significant prognostic factor for OS in patients with MSI-H GC (88) (**Table 3**). In a subsequent study by Jiang et al. (89) involving 879 gastric cancer (GC) patients, it was demonstrated that a higher Immunoscore group was associated with a significant survival advantage in terms of overall survival (OS). The study employed the least absolute shrinkage and selection operator (LASSO) Cox regression model to construct a classifier. The Immunoscore was calculated using a specific formula incorporating the densities of four distinct immune cell types (CD3^+^, CD8^+^, CD45RO^+^, and CD66b) from both the CT and IM regions (89).

Zou et al. (93) conducted a retrospective analysis of 101 GC patients (stage II-III) who underwent gastrectomy followed by chemoradiotherapy. The study aimed to assess the prognostic value of IS, which was determined by IHC staining of CD3^+^ and CD8^+^ T-cell counts in both the CT and IM regions. Based on the IS levels, patients were categorized into three groups. The results revealed that GC patients with higher IS levels exhibited significantly improved DFS (*P-value* < 0.001) and OS (*P-value* < 0.001). The IS demonstrated superior predictive ability compared to the traditional pathological TNM (pTNM) staging system (Area Under the ROC Curve [AUC]: 0.801 vs. 0.677 and 0.800 vs. 0.660 for DFS and OS, respectively) (93).

In a study by Zhang et al. (146), a scoring system incorporated intratumoral and stromal TILs in a cohort of 833 patients with stage I-III GC. The analysis revealed a significant association between TILs and various clinicopathological parameters, including tumor size, histological grade, lymph node metastasis, nerve invasion, tumor thrombus, pathological TNM stage, and World Health Organization subtypes. Moreover, high levels of TILs (hi-TIL) were identified as a positive and significant predictor of OS using Kaplan-Meier survival analysis (*P-value* < 0.001) and multivariate Cox regression analysis (HR = 0.431, 95% CI: 0.347-0.534, *P-value* < 0.001). Additionally, patients with high-TIL tumors demonstrated improved DFS and OS following curative surgery compared to those with low-TIL tumors (146).

Studies have shown that B-cells in the TME have a dual role in promoting or inhibiting tumor growth (147). Consistent with the potential role of B-cells in anti-tumor immunity, Ni et al. (94) conducted a study involving 584 GC patients who underwent radical gastrectomy and found that increased infiltration of CD20^+^ B-cells in GC was independently associated with significantly improved OS and DFS. The study also revealed high CD20^+^ B-cell infiltration levels correlated with lower lymph node metastasis rates and lower pathological TNM stage. Furthermore, both univariate and multivariate Cox regression analyses demonstrated that CD20^+^ B-cell infiltration served as an independent protective factor for prognosis (94).

In a meta-analysis conducted in 2020, it was observed that elevated infiltration of CD3^+^, CD8^+^, and CD4^+^ T-cells within the TME of GC was significantly associated with improved OS outcomes. While it has been postulated that FOXP3^+^ Tregs may induce immune suppression within the TME and consequently exacerbate GC prognosis, the meta-analysis revealed that FOXP3^+^ Treg infiltration did not exhibit a definitive association with clinical outcomes. These findings suggest a potential complex interplay between T-cell subsets and Tregs in the context of GC immunobiology. This necessitates further investigations to elucidate their precise roles and the mechanisms underlying their impact on disease progression (148).

The Klintrup–Mäkinen grade classifies tumor inflammatory cell infiltrates (including the number of lymphoid cells, neutrophilic and eosinophilic granulocytes) at the tumor IM using H&E-stained slides (149). Several studies suggest an association between high Klintrup–Mäkinen grade and good prognosis in colorectal cancer (149, 150). In 2020, Kemi et al. (91) conducted a study to assess and compare the prognostic significance of Immunocore (based on CD3+ and CD8+ lymphocyte densities at the tumor CT and IM) and Klintrup–Mäkinen grades and examine the consistency of Klintrup–Mäkinen grade assessment in GC (91). The study revealed that a high Klintrup–Mäkinen grade independently predicted a longer 5-year overall survival (adjusted HR = 0.59, 95% CI: 0.45–0.77) in both the intestinal (adjusted HR = 0.61, 95% CI = 0.44–0.85) and diffuse subgroups (adjusted HR = 0.52, 95% CI = 0.31–0.86). Both Immunocore and Klintrup–Mäkinen grades emerged as prognostic factors in gastric adenocarcinoma. Moreover, the Spearman correlation coefficient between the three-tiered Immunoscore and the two-tiered Klintrup–Mäkinen grade was found to be 0.425 (91). Recently, Yun et al. (92) established an IS based on the densities of CD3+, CD8+, and Foxp3+ T-lymphocytes in CT and IM regions of 389 patients who underwent surgical resection for stage II/III GC and received adjuvant chemotherapy with 5-FU. They examined the impact of this IS on patient survival. The study found that individuals with a high IS experienced significantly longer DFS (P-value <0.001). Moreover, the IS was consistent between patients with MSI-H and Microsatellite Stable (MSS)/MSI-Low status (83.3% and 80.5%, respectively). Further subgroup analysis based on MSI status revealed that patients with a high IS experienced substantial DFS and OS benefits in both the MSS/MSI-Low group (DFS: HR= 0.527, P-value= 0.004; OS: HR= 0.528, P-value= 0.007) and the MSI-H group (DFS: HR= 0.166, P-value= 0.028; OS: HR = 0.177, P-value = 0.035) (92).

In the study conducted by Yuan et al. (151), the research focus was on establishing connections between blood markers such as lymphocytes, monocytes, platelets, and neutrophils and the primary TME. In order to achieve this, the researchers employed multiplexed IHC to quantitatively assess proteins within the tumor environment at a sub-cellular level in a cohort of 80 GC patients. The study’s findings revealed a significant correlation between a higher lymphocyte-to-monocyte ratio (LMR) at the initial assessment and improved immune-related progression-free survival (PFS), as well as a tendency toward enhanced immune-related OS. Conversely, a higher neutrophil-to-lymphocyte ratio (NLR) was linked to poorer immune-related OS (151).

### 4.4 Colorectal cancer

The relationship between TILs and colorectal cancer (CRC) patient prognosis was first reported in 1998. Specifically, the study demonstrated that the infiltration of CD8^+^ T-cells within cancer cell nests was associated with patient prognosis in the human CRC (152). This finding has since been corroborated by subsequent research and underscores the importance of TILs in progressing and managing CRC (153–155). In a recent study, Xin et al. (106) determined that more CD8^+^ lymphocyte infiltration in either primary tumors or paired distant metastases predicted an excellent prognosis (*P-value* =0.036 and 0.031, respectively). In multivariate analysis, CD8^+^ TIL density in primary tumors was an independent predictive factor for OS (HR= 0.28, 95% CI= 0.09-0.93, *P-value* =0.038) (106).

Elomaa et al. (105) conducted a study to assess the prognostic significance of the spatial distribution of T-cells in CRC. The study involved using IHC and digital image analysis to identify CD3^+^ and CD8^+^ cells and tumor cells in a total of 1229 CRC samples. The authors introduced the T-cell proximity score as a novel prognostic parameter based on evaluating the co-localization of tumor cells with T-cells. The study’s findings demonstrated that a high T-cell proximity score was significantly correlated with favorable outcomes in CRC. Importantly, this association remained significant even after accounting for potential confounding factors such as disease stage, MMR status, and T-cell density score (105) (**Table 4**). These results suggest that the spatial arrangement and proximity between T-cells and tumor cells could be a valuable prognostic factor in CRC.

Immunotherapy with ICIs has demonstrated clinical benefits in colon cancer patients, particularly those with microsatellite MSI-H status. In a clinical study conducted in 2020, pembrolizumab, an anti-PD-1 agent, exhibited significant improvements in PFS (16.5 vs. 8.2 months) compared to standard treatments as a first-line therapy for metastatic MSI-H colon cancer patients (156). Although MSI-H has shown promise as a predictive biomarker for immunotherapy across pan-cancer, its clinical applicability in colorectal cancer is limited by its relatively low prevalence among CRC patients. Additionally, some colon cancer patients with MSI-H status may still exhibit intrinsic or acquired resistance to immunotherapy (157). Therefore, the efficacy of MSI status as a biomarker for immunotherapy in colon cancer patients may be limited, and alternative or complementary biomarkers should be explored. Extensive lymphocytic infiltration is more frequently observed in MSI tumors than in MSS tumors. The relationship between TILs and MSI status can provide further insight into differentiating CRC patients with better prognostic outcomes. Understanding this relationship may help identify additional factors for predicting prognosis and response to immunotherapy in colon cancer patients.

In line with the concept mentioned above, Williams et al. (96) developed a TIL/MMR-based classification system to stratify the prognosis of CRC subtypes in patients with stage II/III tumors. Interestingly, the study found that even in the presence of MSI, a TIL-low status was associated with a clinically aggressive phenotype (96). This suggests that TIL status can provide additional prognostic information beyond MSI status alone and may help identify patients with poorer outcomes despite having MSI-positive tumors.

Indeed, immunotherapies have demonstrated limited efficacy in the treatment of MSS and MMR-proficient (pMMR) metastatic colorectal cancer (mCRC) patients (158). However, within the subset of MSS mCRC patients, evidence indicates that a higher baseline density of CD8+ TILs is associated with an increased likelihood of benefiting from immunotherapy. Specifically, a study involving durvalumab (anti-PD-1) in combination with trametinib (a mitogen-activated protein kinase inhibitor) demonstrated that MSS mCRC patients with higher levels of CD8+ TILs at baseline were more likely to experience positive treatment responses or clinical benefits (103).

In a study of neoadjuvant immunotherapy (nivolumab [anti-PD-1] plus ipilimumab [anti-CTLA-4]) for stage I-III colon cancer patients, CD8^+^PD-1^+^ T-cell infiltration was a predictive biomarker of response in pMMR patients (97).

In a single-arm phase 2 clinical trial conducted by Kuang et al. (104), the researchers investigated the potential of concurrent treatment with the DNA methyltransferase inhibitor azacitidine to enhance the antitumor activity of pembrolizumab in mCRC patients. The trial enrolled 30 patients who were refractory to chemotherapy, and the participants received a median of three cycles of the combined therapy. Notably, the density of CD8^+^ TILs increased during the treatment compared to the levels observed before treatment initiation. Furthermore, a higher baseline CD8^+^ TIL density was associated with a greater likelihood of benefiting from the treatment. In addition, the study also observed a correlation between tumor demethylation during treatment and tumor CD8^+^ TIL density increases (104). This suggests that the demethylation process may play a role in modulating the immune microenvironment by influencing the density of CD8^+^ TILs.

In an open-label, single-center pilot trial, combination therapy of perioperative durvalumab and tremelimumab (an anti-cytotoxic T lymphocyte-associated antigen 4 antibody) was investigated as a potential treatment for patients with resectable liver metastasis CRC—the trial aimed to assess whether this combination therapy could enhance immune responses in this disease setting. The study enrolled 24 patients, and the findings showed that four of them achieved a complete pathological response. Among them, two had dMMR status, and the other two had POLE (DNA polymerase ϵ) mutations. Pre- and post-treatment tumor tissue analysis indicated comparable levels of T-cell infiltration, but there was evidence of CD8^+^ and CD4^+^ activation after treatment. Also, post-treatment samples from patients with prolonged RFS showed increased B-cell transcriptome signature and B-cell density (102).

Changes in the epigenetic makeup of TILs have been linked to how cancer patients respond to immunotherapy. More precisely, modifications in DNA methylation and histone structure can impact the activation and functioning of T-cells, potentially affecting how well TILs can identify and eradicate cancer cells (159). Zou et al. (101) devised a DNA methylation signature tailored for CD8+ TILs to assess immune response and prognosis in CRC. Using Illumina EPIC methylation arrays, they identified specific DNA methylation patterns in CD8+ T-cells and created a signature score. Their study revealed that a low CD8+ MeTIL score, which signifies an abundance of CD8+ TILs, was linked to MSI-H tumors and predicted improved survival among CRC cohorts. These findings suggest that the CD8+ MeTIL signature score could be a valuable prognostic biomarker for CRC, highlighting the potential of epigenetic signatures in assessing immune response and prognosis in cancer (101).

Consensus molecular subtypes (CMS) is a classification system that stratifies CRC into four distinct subtypes based on gene expression profiling, providing insights into the tumors’ underlying biology and clinical behavior (160). The CMS classification system has been shown to have prognostic and predictive value and may help guide treatment decisions and improve outcomes for patients with colorectal cancer (161). In a recent study, Hu et al. (162) conducted a comprehensive analysis of CMS subtypes’ molecular characteristics and immunotherapy responses using multiple bioinformatics databases. Their findings suggest that CMS1 patients are more likely to respond positively to immunotherapy than the other CMS subtypes. This is attributed to the presence of immune infiltration and activation, which is significantly higher in the CMS1 subtype than in the other subtypes. In particular, TILs were found to be significantly more abundant in the CMS1 subtype (163). These results provide important insights into the potential use of CMS subtyping to predict immunotherapy response in colorectal cancer patients.

**Figure 6:**
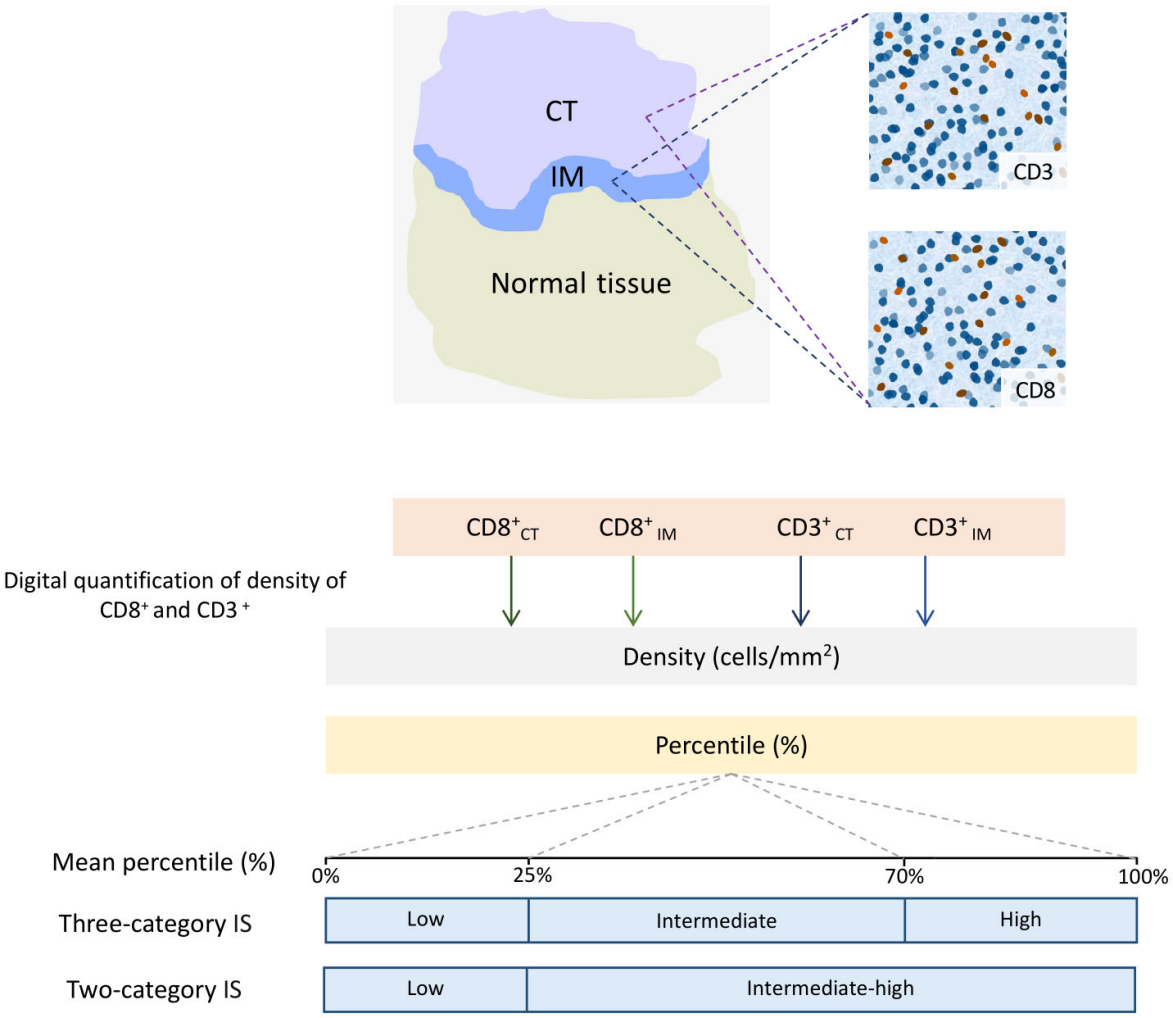
Schematic Illustration of the Immunoscore (IS) determination. In the top left image, digital pathology software is used to automatically identify tumor (CT) tissue, invasive margin (IM), and normal tissue in colon cancer samples. In the top right images, the software also automatically detects the numbers of CD3^+^ and CD8^+^ T-cells. The bottom chart illustrates the calculation of the IS for colon cancer. The method involves converting the densities of CD3^+^ and CD8^+^ T-cells in both the CT and IM into percentile values. Then, the means of four percentiles are calculated for the IS. In a three-category IS analysis, the mean densities within the ranges of 0-25%, 25-70%, and 70-100% are categorized as “low,” “intermediate,” and “high,” respectively. In a two-category analysis, the mean densities between 0-25% are labeled as “low,” while densities ranging from 25-100% are classified as “intermediate-high.”

ImmunoScore^®^ (IS^®^) is a novel diagnostic tool that has gained attention in recent years to predict the risk of cancer recurrence in patients with CRC. Developed by the Society for Immunotherapy of Cancer, IS^®^ measures the density of immune cells in tumor tissue, providing valuable information about the patient’s immune response to the cancer. The use of IS^®^ has been shown to be an effective means of predicting the risk of recurrence in CRC patients, making it a promising tool in the fight against this deadly disease (164). IS^®^ quantified the density of two types of immune cells, CD3^+^ and CD8^+^ T-cells, in the CT and IM for each case, using a standardized IHC staining protocol. The stained tissue samples are then scanned and analyzed using digital pathology software to generate a score reflecting the immune cells’ density. Next, the means of four percentiles (two markers and two regions) are calculated and converted into the IS^®^. In a three-category IS^®^ analysis, TIL densities between 0-25%, 25-70%, and 70-100% are scored as “low,” “intermediate,” and “high,” respectively. In a two-category analysis, TIL densities between 0-25% are scored as “low,” while densities between 25-100% are scored as “intermediate-high” (165) (**Figure 6**).

Several studies have reported the validity of IS^®^as a prognostic marker in patients with CRC, regardless of the type of chemotherapy regimen used in the treatment (166, 167). In 2018, the Society for Immunotherapy of Cancer aimed to validate the Consensus IS^®^’s accuracy and prognostic value in classifying CRC patients. The study enrolled 2,681 stage I-III colon cancer patients from 14 different international centers. The IS^®^ was determined by quantifying the density of CD3^+^ and CD8^+^ T-cells in the tumor core and IM using a standardized protocol. The study found that the patients with a high IS^®^ had the lowest risk of recurrence at five years vs. patients with a low IS^®^ (HR= 0.20, 95% CI= 0.10-0.38; *P-value <*0·0001) (164). Two years later, the Society for Immunotherapy of Cancer conducted another multicenter study to explore the association between the IS^®^ and the effect of chemotherapy on time to recurrence (TTR) in patients with stage III colon cancer. As expected, the study found that patients with a high IS^®^ had a significantly prolonged TTR, OS, and DFS (all *P-values* < 0.001). Among patients with MSS tumors, the high IS^®^ was significantly associated with prolonged TTR (HR= 0.36; 95% CI= 0.21-0.62; *P-value* = 0.0003). Although, in the high-IS^®^ group, chemotherapy was significantly associated with survival for both low-risk [HR (chemotherapy vs. no chemotherapy)= 0.42; 95% CI= 0.25-0.71; *P-value* = 0.0011] and high-risk [HR (chemotherapy vs. no chemotherapy)= 0.5; 95% CI= 0.33-0.77; *P-value* = 0.0015] patients; this association was not observed for the low-IS^®^ group (*P-value* > 0.12) (99). These results support the implementation of the consensus IS^®^ as a new component of a TNM-Immune cancer classification.

In a study by Pagès et al. (98), the three-category IS^®^ was used to investigate its efficacy in predicting response to oxaliplatin-based adjuvant chemotherapy in stage III CRC patients. The study found that patients with a low IS^®^ were at a higher risk of relapse or death compared to those with an intermediate-high IS^®^ (HR= 1.54; 95% CI= 1.24-1.93, *P-value* = 0.0001). The IS^®^ remained significantly associated with DFS in multivariable analysis when adjusted for gender, histological grade, T/N stage, and MSI (*P-value* = 0.003). In patients treated with mFOLFOX6 [leucovorin calcium (folinic acid), fluorouracil, and oxaliplatin], a statistically significant interaction was observed between IS^®^ and treatment duration (3 vs. 6 months) in terms of predicting DFS (*P-value* = 0.057) (98).

Collectively, these findings suggest that the IS^®^ may be a valuable prognostic biomarker for predicting patient outcomes and guiding treatment decisions in colon cancer. In addition, these studies provide strong evidence for implementing the consensus IS^®^ as a new component of a TNM-Immune classification of cancer. The standardized protocol used in the studies may also pave the way for further international collaborations and standardization of immune biomarkers in cancer research.

### 4.5 Hepatocellular carcinoma

Hepatocellular carcinoma (HCC) represents a significant global health burden, accounting for the third-highest cancer-related mortality worldwide (1). Recent advances in managing HCC introduced new therapeutic drugs, such as Lenvatinib, as a first-line therapeutic agent in unresectable HCC patients (168). However, the prognosis of HCC cases is poor, and the rate of recurrence and metastasis is still high (169, 170). Anticipation of discovering new predictor biomarkers in survival and treatment response of HCC patients and generating personalized treatment strategies will be increased in the near future.

Several recent studies have assessed the role of CD8^+^ T-cells as a significant tumor-infiltrating lymphocyte in the prognosis of HCC. The result was controversial; Sun et al. showed that high levels of CD8^+^ are associated with better prognosis (171) (**Table 5**). However, two other studies found no relation or even worse association between CD8^+^ density and the survival of patients (172, 173). Stulpinas et al. hypothesized that the region of assessment of CD8^+^ density in HCC samples can affect the results. Infiltration of lymphocytes in tumor microenvironment and non-tumoral nearby parenchyma can independently manage the survival of patients (110). In this study, the samples of 106 patients with HCC underwent H&E staining and hexagonal grid-based digital image analysis. The density of CD8^+^ T-cells was measured in malignant and non-malignant regions of the samples. Outcomes revealed that an increase in the standard deviation of CD8^+^ density in tumors is positively associated with better OS (HR= 0.41, *P-value*= 0.0026). In apposition, higher mean CD8^+^ density in non-tumoral parenchyma is an independent factor in worsening OS (110). Interestingly, in the next step, the authors create a new score for the prognosis of HCC patients by measuring another parameter. The combined OS risk score consists of 5 items. A higher score is correlated with intravascular invasion of the tumor, long duration of surgery, blood Basophil count > 0.055 × 109/L, and aspartate transaminase (AST) level > 135 U/L, which causes worsened OS. Also, stage pT1 of HCC, bigger tumor size, higher mean CD8^+^ density in the non-malignant region, wider tumor-free margin, and shorter time of surgery are associated with shorter regression‐free survival (110).

In another theory, the controversial effect of CD8^+^ T-cells in the prognosis of cancer can result from regulatory cells in the tumor microenvironment, which can affect the function of CD8^+^ T-cells (174). Exhausted CD8^+^ T-cells lose their effective cytotoxic capacity. Also, cytokine secretion and proliferative ability of the T-cell is decreased (175). Exhaustion in CD8^+^ T-cell causes expression of inhibitory receptors such as PD1 and T-cell immunoglobulin and mucin-domain-containing-3 (TIM3) (176). Despite advances in PD-1 blocked therapy in different cancers, the optimal response still depends on T-cell infiltration and function in the tumor environment (177). As a result, discovering the proteins and cells that can affect the CD8^+^ T-cell function can help to increase the quality of PD-1 blocked therapy.

In a recent study, Wang et al. assessed the expression level of thymocyte selection-associated high mobility group box protein (TOX) in 40 specimens of HCC (107). The role of TOX in T-cell differentiation has been proved in studies (107, 178). Wang et al. divided the HCC samples into three groups for evaluating the CD8^+^ T-cell, including good effector function (PD-1^-^TIM3^-^), moderate exhaustion (PD-1^int^ TIM3^+^) and severe exhaustion (PD-1^hi^ TIM3^+^). Under transcriptome sequencing analysis, the expression of TOX was higher in the group of PD-1^hi^ TIM3^+^ CD8^+^ T-cells (107). Additionally, the knockdown of the TOX gene is associated with better anti-tumor function of CD8^+^ T-cells. In fact, TOX promotes PD-1 recycling on CD8^+^ T-cells through inhibiting RAS and PI3K-Akt pathways, which hinder the lysosomal digestion of PD-1 on CD8^+^ T-cells (107). In addition, lower levels of TOX on CD8^+^ T-cells in the periphery are associated with better prognosis and lower TNM stages in HCC patients (107). These findings open new insights into improving the PD-1 blocked therapy by downregulating TOX expression, which enhances CD8^+^ T-cells from exhaustion level.

Another way to increase the quality of PD-1 blocked therapy is to select co-stimulatory receptors, which enhance T-cell differentiation and cytotoxic functions (179). Kim et al. showed that CD137L is one of the most co-stimulatory receptors expressed in HCC. This study was performed on 79 patients with HCC and demonstrated that CD137L expression is higher in PD-1^high^ CD8^+^ T-cells (108). To evaluate the effect of CD137L expression on PD-1^high^ CD8^+^ T-cells function, the amount of CD39^+^CD103^+^ CD8^+^ TIL subsets was measured and demonstrated that an increased level of CD39^+^CD103^+^ is observed in CD137L positive cells, which is associated with activation of T-cells and tumor responsiveness. Besides, the CD137L expression on PD-1^high^ CD8^+^ T-cells renovates the potency and proliferation of T-cells (108). *In vitro* assay of CD137L agonistic antibody effect on CD8^+^ TILs demonstrated a notable increase in proliferation of CD8^+^ T-cells and production of IFN-γ and TNF-α. Kim et al. proved that combination therapy of T-cells with 4-1BB co-stimulatory agonists and PD-1 blocked can improve the strength of T-cells in HCC (108).

Altogether, finding new pathways of markers that affect the T-cell Exhaustion and regulate their expression besides PD-1 blocked therapy can provide new promising immunotherapeutic strategies in managing HCC.

Advances in immune checkpoint inhibitor therapy, such as PD-1 blockade and targeted molecular therapy, have unveiled novel systematic approaches to anti-cancer treatment for HCC patients. Finn et al. proved that combination therapy with Atezolizumab (anti-PD-L1) plus Bevacizumab (anti-VEGF monoclonal antibody) increases OS and PFS of HCC patients and replaced as the primary chemotherapy line in advanced HCC patients (180). Despite this, HCC is still a poorly controlled cancer, and new personalized therapeutic strategies are needed. Kuwano et al. measured CD8^+^ T-cell as a predictive marker for progression-free survival in patients who underwent Atezolizumab plus Bevacizumab and Lenvatinib alone treatments. Lenvatinib is a molecular inhibitor of multiple receptor tyrosine kinases such as fibroblast growth factor (FGF), VEGF, and platelet-derived growth factor (PDGF) receptors. It is one of the first-line choices in treating unresectable HCC (181). Computed tomography (CT) or magnetic resonance imaging (MRI) were tools used in this study to evaluate the response to treatment every 6 to 12 weeks. Immunohistochemistry of CD8^+^ T-cells of HCC biopsy in 24 patients before treatment with atezolizumab plus bevacizumab demonstrated that higher CD8^+^ T-cells are associated with better progression-free survival. However, no correlation has been found between high CD8^+^ T-cell density in tumoral biopsy and Lenvatinib alone treatment (111). This study suggests a personal therapeutic strategy in HCC patients, though tumor liver biopsy is invasive. Nevertheless, new non-invasive strategies are needed to find markers for predicting treatment response in HCC patients.

The existence of TILs in the tumor microenvironment of the cancer is an essential component for the response and activation of immunotherapeutic agents (182)

Gao et al. showed that evaluating the prognosis of overall immune markers of TILs can bring newer approaches in HCC management and eliminate the limitation and biased information of valuing the subpopulations of TILs individually (109). This study was accomplished in two phases. The first phase was estimating the TILs density in 315 samples of HCC patients by H&E staining, which is called WCH set, and the next phase was validating the prognosis of TILs in 370 HCC patients from The Cancer Genome Atlas (TCGA). The tumors were categorized into three groups: high TILs ≧ at 50%, intermediate TILs between 10% and 50%, and low TILs < 10%. The results prove that OS and DFS is better in higher (OS, HR= 0.33 (0.13-0.83), *P-value*= 0.02, DFS, HR= 0.21 (0.09-0.52), *P-value*= 7.86 × 10-4) and intermediate (OS, HR= 0.54 (0.34-0.86), *P-value*= 0.01, DFS, HR= 0.34 (0.21-0.56), *P-value*= 1.56 × 10-5) TILs groups in WCH set. Also, this data is validated in the TCGA set, confirming that low TIL density was associated with worse OS and DFS. In conclusion, the result of this study suggests a promising approach to using TIL density for determining the prognosis of HCC patients in clinical assessments (109).

## 5 Future directions toward precision medicine

There are a considerable number of studies on the function of TILs in GI tract cancers, which fully show an active immune response correlates with survival and provides a rationale for TIL therapy in GI cancers. The intricate interplay between TILs and tumor cells in TIME has been demonstrated to be a key determinant of the response to immunotherapy. Currently, PD-L1 expression, tumor mutation burden (TMB), and MSI-H/dMMR are the sole predictive biomarkers to determine eligibility for treatment with ICI, yet they lack robustness (183).

Considering the previously discussed concept, the assessment of TILs can offer valuable insights into tumor immune response and potentially serve as an additional parameter for evaluating treatment outcomes. Considering TILs as a predictive factor for response to neoadjuvant therapy in preoperative prognostication could be a significant advancement toward personalized treatment approaches. In certain cancer types, biopsies have been effectively employed to assess TILs as predictors of treatment response (184, 185). This demonstrates the potential utility of TIL evaluation in the clinical practice of GI cancer patients, allowing for identifying patients who are more likely to respond favorably to specific therapies, thereby enabling tailored treatment strategies.

In the wake of an immunotherapy revolution and given the prognostic promise of the Immunoscore classification system in various cancers. Perhaps the Immunoscore^®^ for colorectal cancer was the most successful one among all human cancers, which has demonstrated significant potential as an adjunct or alternative to the TNM cancer staging system. Therefore, the International Immunoscore Project has attempted to standardize immune measurements in other GI cancers (4). Notwithstanding encouraging evaluation of its efficacy in CRC, some studies addressed some shortfalls of the IS system for other cancers. For instance, in EC, a study utilizing a similar approach as Immunoscore^®^ failed to detect significant associations between patient survival and the expression of CD3^+^, CD8^+^, or CD45RO^+^ (5). IS has been subject to scrutiny in other types of cancer, with several studies raising concerns about its failure in efficacy for not considering other immune cell infiltrates, including macrophages and NK cells, as well as the heterogeneity of T-cell infiltrates within the TIME (6).

Indeed, the individual lymphocyte subsets within the TME are crucial but insufficient for effective tumor immune control. The success of an antitumor immune response depends on various factors, including the proper localization of TILs, clustering, interplay, and costimulation of all lymphocyte subsets. These subsets coordinated, and synergistic action contributes to an effective immune response against tumors. Proper identification of the presence and location of TILs within the tumor tissue and their distribution and proximity to tumor cells can provide valuable information for predicting prognosis and therapy response.

This may suggest that a more extensive IS may be required for other GI cancers compared to what is currently proving successful for CRC. Among others, Wen et al. (7) have proposed a novel four-score system for GC that integrates the expression of CD8^+^, PD-L1 on tumor cells and immune cells, and PD-1^+^ on immune cells. This approach exhibits potential for superior prognostic application compared to existing models for GC patients. There is another challenge to incorporating the assessment of TILs into routine clinical practice for GI cancer oncology and pathology reporting due to the existence of several distinct methods for histological quantification of TIL subsets, each with its own specific scoring technique or cut-off. To establish reference values and confirm the validity of such approaches, extensive homogenous comparative analyses are necessary prior to the routine implementation of TIL assessment in the pathology of GI cancers. Moreover, Artificial intelligence (AI) is poised to transform the field of pathology by enabling more efficient and accurate diagnoses, as well as more personalized treatment plans. By leveraging machine learning algorithms to analyze large datasets and identify subtle patterns, AI has the potential to improve the accuracy and speed of pathology diagnoses, ultimately leading to better patient outcomes (186). Automatic digital machine learning presents a promising tool for assessing TILs precisely and consistently. This advanced technology allows for the simultaneous evaluation of complex TIL composition and localization using multiple defining markers while minimizing interobserver variability commonly encountered with manual assessment techniques (187). The use of an automated signature of CD8xPD-L1 has been reported as a predictive marker in non-small-cell lung cancer patients (188). However, further investigation is required to determine the optimal implementation and integration of digital analysis as an independent technology into daily clinical practice to fully leverage its potential benefits (186). A recent report demonstrated the agreement between manual and computational scoring of TILs. Yoo et al. (189) developed an open-source software-based analytic pipeline to quantify TILs from whole-slide images of 578 stage III or high-risk stage II CRC patients, which were stained for CD3^+^ and CD8^+^ using IHC. The findings indicated acceptable concordance between the automatic quantification of TILs and visual inspection by a pathologist, supporting the reliability of the computational approach (189). A semi-automated approach may be the most effective to optimize the application of digital analysis findings. In this method, a pathologist first selects the area of interest to ensure that a representative field is evaluated. Subsequently, a computer application or software is utilized to identify and provide an estimation of TILs in the selected area. The recent introduction of an open-source algorithm for the automated evaluation of TILs in melanoma is a notable development that has generated enthusiasm among researchers (190). This innovative tool has the potential to significantly advance the field of digital pathology by streamlining the evaluation process and improving the accuracy and reliability of TIL assessment. Finally, adopting a universally established method and cutoff for evaluating TILs of each cancer could make the quantification process more effective and promote their clinical implementation. This approach would enhance the consistency and reproducibility of TIL assessment across different laboratories and improve their clinical relevance and utility in cancer diagnosis and treatment. Moving on from the role of TILs in forecasting the prognosis into therapeutic utilities, we postulated that combinatorial immunotherapy regimens represent a pivotal approach for optimizing response rates and clinical outcomes in patients undergoing immunotherapy for cancer treatment. Specifically, the concurrent targeting of T-cells and NK cells through ICIs has garnered attention and is currently being translated into clinical practice. Notably, emerging agents such as anti-NKG2A (anti-CD94/NK group 2 member A) and anti-TIGIT (anti-T-cell immunoreceptor with immunoglobulin and ITIM domains) are being investigated in this context (191, 192).

Another future direction for TIL-based personalized medicine is the development of novel therapeutic strategies that exploit the unique properties of TILs. For instance, recently, a novel approach to immunotherapy known as adoptive cellular therapy (ACT) has emerged, offering a potential solution to the problem of drug resistance observed in solid tumors treated with ICI. This innovative approach involves isolating and expanding immune cells, such as T-cells, from a patient’s tumor or blood and their subsequent infusion back into the patient to enhance the immune response against the cancer (193). TIL therapy has been proven to have impressive clinical benefits for patients with various solid tumors such as lung cancer (194), cervical cancer (195), melanoma (196), CRC (197), and cholangiocarcinoma (198); regarding CRC, in phase two clinical trial involving a 50-year-old woman with metastatic colorectal cancer, a single infusion of 1.48 × 10^11^ TILs was administered. The infusion consisted of approximately 75% CD8^+^ T-cells specifically engineered to recognize the KRAS G12D mutant. Following treatment, all metastatic lesions in the patient regressed, and a nine-month partial response was achieved (197). Another clinical trial of TIL therapy using CAR (chimeric antigen receptor) cells targeting MAGE-A4 in patients with advanced solid tumors showed that patients with EC experienced an objective partial response in mediastinal and para-esophageal lymph nodes (199). These promising results suggest that TIL therapy may have significant potential as a treatment for solid cancers, and further research in this area is warranted for other GI cancers.

## 6 Conclusion

Immunotherapies have made remarkable strides, but there are still challenges to overcome. Limited response rates, unpredictable efficacy, and potential side effects have hindered their clinical application. However, by understanding how cancer cells and TILs interact in a spatial coordinate system, we can gain new insights into cancer progression and improve the efficiency of current immunotherapies. Thanks to new technologies emerging in the field of digital machine learning, with a focus on spatial mapping and quantification, the systematic and comprehensive understanding of *in situ* crosstalk between immune cells and cancer cells in the TME and their dynamic changes during treatment is within reach. These advancements are sure to propel the clinical success of immunotherapies even further.

## Author contributions

MoeP: Supervision, Writing – original draft, Writing – review & editing. YG: Conceptualization, Writing – original draft. MobP: Visualization, Writing – original draft. ES: Writing – review & editing. EN-M: Conceptualization, Project administration, Supervision, Writing – review & editing.
